# Genome‐wide association meta‐analyses identify novel genetic risk loci and polygenic phenotype associations for heroin, methamphetamine and alcohol dependences

**DOI:** 10.1002/ctm2.659

**Published:** 2022-01-24

**Authors:** Su‐Hua Chang, Yan Sun, Fan Wang, Xiang‐Wen Chang, Ying‐Jian Zhang, Tian‐Ye Jia, Hong‐Qiang Sun, Wei‐Hua Yue, Ping Wu, Lin Lu, Jie Shi

**Affiliations:** ^1^ NHC Key Laboratory of Mental Health (Peking University) National Clinical Research Center for Mental Disorders (Peking University Sixth Hospital) Chinese Academy of Medical Sciences Research Unit (No.2018RU006) Peking University Institute of Mental Health Peking University Peking University Sixth Hospital Beijing China; ^2^ National Institute on Drug Dependence Peking University Beijing China; ^3^ Beijing Key Laboratory on Drug Dependence Research, Peking University Beijing China; ^4^ Beijing HuiLongGuan Hospital, Peking University HuiLongGuan Clinical Medical School Beijing China; ^5^ Social, Genetic and Developmental Psychiatry Centre Psychology & Neuroscience King's College London De Crespigny Park Institute of Psychiatry London UK; ^6^ Institute of Science and Technology for Brain‐Inspired Intelligence, Ministry of Education‐Key Laboratory of Computational Neuroscience and Brain‐Inspired Intelligence and Research and Research Institute of Intelligent Complex Systems Fudan University Shanghai China; ^7^ The State Key Laboratory of Natural and Biomimetic Drugs Peking University China; ^8^ The Key Laboratory for Neuroscience of the Ministry of Education and Health Peking University China

Dear Editor,

This work presented a significant correlation between heroin and methamphetamine dependence (HD and MD) which distinguished with alcohol dependence (AD) at the genome‐wide level. Three novel risk loci were identified for HD and MD. The shared polygenic risk with cognition and attention‐deficit hyperactivity disorder (ADHD) further profiled the similar genetic characteristics between HD and MD compared to AD.

Substance dependencies (SD) are one of the leading public health concerns worldwide. Drug markets are in a constant state of flux. The combined use of different addictive substances, especially illegal drugs, is common among addiction patients. Genetic factors contribute to approximately 40%–70% of the variance in persistent SD.^1^ Capturing the shared and specific genetic mechanism of different substance dependences is crucial for coping with the changeable types of addiction.

In this study, genome‐wide association meta‐analyses (GWMA) were performed independently for HD, MD and AD based on two data sets (DS) of substance‐specific dependence patients (1028 HD, 1750 MD, 537 AD and 2862 shared controls for DS1, 980 HD, 701 MD, 224 AD and 1111 shared controls for DS2) (Supplementary Tables S1 and S2, Supplementary Figures S1 and S2, supplementary methods). Consist with our previous study in DS1^2^, One significant locus at chr12 *ANKS1B* was identified for both HD (peaked in rs112706556, *p*_meta_ = 4.99e‐8) and MD (peaked in rs58720542, *p*_meta _= 4.401e‐9) (Table 1, Figure 1A and B); two well‐known loci in chr4 *ADH* cluster and chr12 *ALDH* cluster were verified for AD (Supplementary Figure S3). Genetic correlation analysis using LD score regression^3^ showed MD and HD were significantly correlated (*r_g _* = 0.8618, *p *= 7.361e‐5), but not for HD and AD, MD and AD. The cross‐dataset pairwise polygenic risk score (PRS) analysis further validated these relationship patterns (Supplementary Table S3).

**TABLE 1:** Association results for the three significant loci for HD&MD and related functional mapping and annotation

GWAS Lead SNP	Predicted genes / Genomic coordinate (hg19) /A1:A2	Substance dependence trait	Beta	SE	*p* Value of GWAS meta	#SNPs in LD (*r* ^2 ^> 0.6)	SNPs in LD with CADD score > 12.37^a^	SNPs in LD with RegulomeDB scores < 5	Chromatin state^b^
rs112706556	ANKS1B (intronic)/ chr12:99839855‐99919728/ A:G	AD	0.1489	0.03	0.3232	35	rs7313882 (*p* = 9.334e‐9, CADD = 14.22); rs7962904 (*p* = 1.207e‐8, CADD = 12.73)	3 SNPs with score = 3a, 3 SNPs with score = 4	Enhancer, strong transcript, heterochromatin
		HD	0.6107	0.112	**4.99E‐08**				
		MD	0.4867	0.0859	**1.46E‐08**				
		HD&MD	0.4611	0.0801	**8.70E‐09**				
rs74330628	RPL7P13 (upstream of TSS), NRXN1 (19 kb downstream)/ chr2:50023593‐50126480/A:G	AD	0.1684	0.1721	0.328	30	rs11675829 (*p* = 8.201e‐6, CADD = 15.82); rs60582193 (*p* = 5.847e‐4, CADD = 16.09)	1 SNP with score = 3a	Active TSS, enhancer, strong transcript, heterochromatin
		HD	–0.5038	0.1251	5.68E‐05				
		MD	–0.4834	0.0959	4.64E‐07				
		HD&MD	–0.489	0.0889	**3.84E‐08**				
									
rs76965632	GTF2IRD1 (intronic)/ chr7:73898830‐73938239/ C:T	AD	–0.5245	0.3531	0.1374	3	NA	NA	Enhancer, strong transcript, active TSS
		HD	–1.1865	0.2373	5.73E‐07				
		MD	–1.0607	0.2032	1.78E‐07				
		HD&MD	–1.0578	0.1865	**1.41E‐08**				

AD: Alcohol dependence; HD: heroin dependence; MD: Methamphetamine dependence; HD&MD: combined heroin dependence and methamphetamine dependence; NA: not available. The GWAS meta *p* value < 5.0e‐8 is marked with bold.

^a^ The *p* value is the *p* value of the SNP in the GWAS meta‐result. CADD > 12.37 has been suggested as the minimum value for pathogenic SNPs and has been used as a threshold for highly deleterious SNPs.^10^ ^.^

^b^ Chromatin state analysis in neuronal cell lines/tissues, including E007, E009, E010, E053, E054, E067, E068, E069, E070, E071, E072, E073, E074, E081, E082, E125 in Roadmap Epigenomics.

**FIGURE 1:**
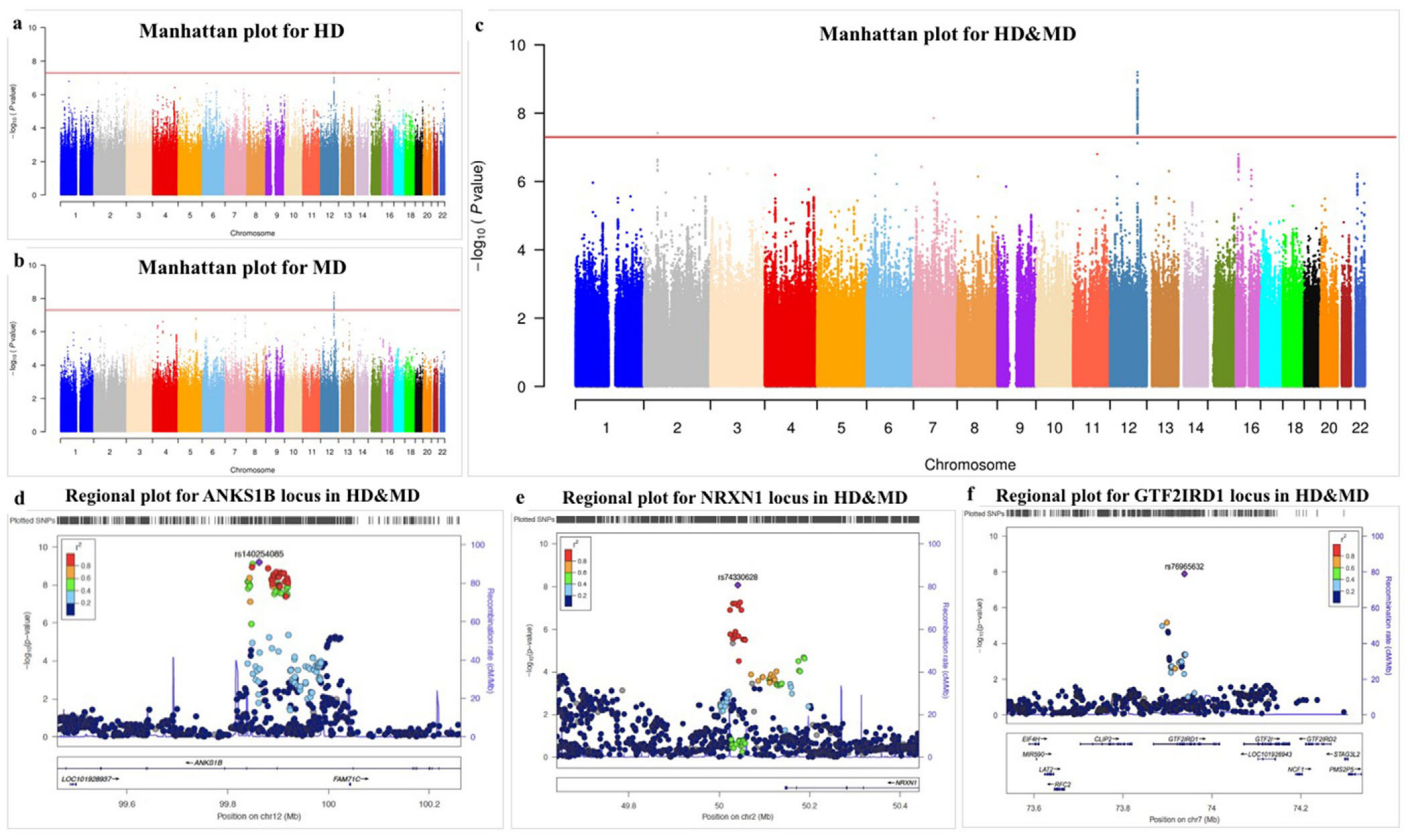
Manhattan and regional plots for drug dependence traits. (A) Manhattan plot for HD. (B) Manhattan plot for MD. (C) Manhattan plot for HD&MD. The red line denotes the threshold of *p *< 5e‐8. (D)–(F) The regional plots for the three significant loci of HD&MD in chr12 (D), chr2 (E) and chr7 (F)

Based on the high genetic correlation between HD and MD, the combined GWMA for HD&MD versus controls were analysed, in which HD and MD were grouped as cases and compared with controls (Table 1 and Figure 1). In addition to the *ANKS1B* locus (peaked in rs140254085, *p*_meta _= 6.355e‐10), another two novel loci located in chr2 locus (peaked in rs74330628 downstream of *NRXN1*, *p*_meta _= 3.84e‐8) and chr7 locus (peaked in *GTF2IRD1* intron rs76965632, *p*_meta _= 1.41e‐8) were identified (Supplementary Table S4). The functional annotations for the three significant loci are shown in Table 1. Several regulatory features were located in the loci (Supplementary Figure S4), and eQTL data showed the loci regulated the expression of *ANKS1B* and *NRXN1* in brain tissues (Supplementary Table S5, Figure 2A and Supplementary Figure S5). By comparison, the three significant loci of HD&MD were not associated with AD, while the chr4 *ADH* and chr12 *ALDH* loci were not associated with HD and MD or had a different direction in HD and MD with AD (Supplementary Figure S6). These findings are consistent with previous twin study that showed a closer genetic correlation across illicit drug dependences compared to alcohol dependence.^4^

**FIGURE 2:**
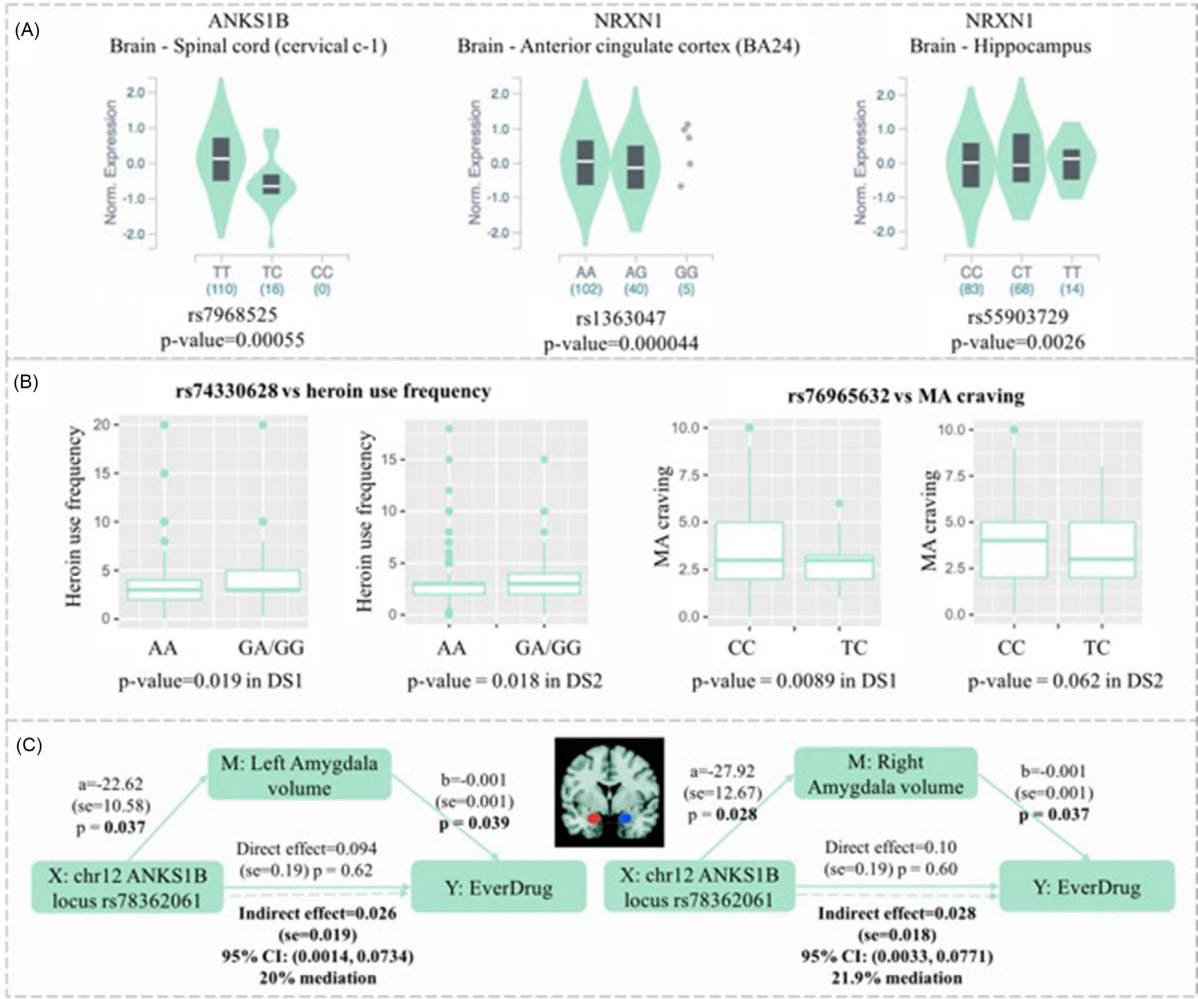
Function of the significant loci. (A) eQTL data for the significant loci in GTEx. SNP rs7968525 was associated with the expression of *ANKS1B* in the brain–spinal cord (cervical c‐1), SNP rs1363047 was associated with the expression of *NRXN1* in the brain–anterior cingulate cortex (BA24) and SNP rs55903729 was associated with expression of *NRXN1* in the brain–hippocampus. (B) Association of the significant loci with heroin use frequency and MA craving. Rs74330628 in the *NRXN1* locus was associated with heroin use frequency, rs76965632 in the *GTF2IRD1* locus was associated with MA craving. (C) Mediation model used SNP rs78362061 of the chr12 *ANKS1B* locus as *X*, left/right amygdala as *M* and EverDrugs as *Y* by using the Human Connectome Project data. SNP rs78362061 of the *ANKS1B* locus was associated with left amygdala volume (*p* = .037) and right amygdala volume (*p* = .028), which were also associated with EverDrugs (*p* = .003 for the left amygdala, *p* = .037 for the right amygdala). SNP rs78362061 of the *ANKS1B* locus had an indirect effect on EverDrugs through the mediation of the left amygdala (20%) and right amygdala (21.9%)

Next, the association with addiction characteristics of the significant loci for HD&MD was examined (Supplementary Table S6 and Figure 2B). The protective allele (A) of rs74330628 in the *NRXN1* locus was associated with a lower frequency of heroin usage (*p*_adj _= .0167). The protective allele (C) of rs76965632 in the *GTF2IRD1* locus was negatively associated with craving of MA (*p*_adj_*__* = .0256). Using the Human Connectome Project data, the SNPs of *ANKS1B* was associated with risk of ever used illicit drug (EverDrugs) (Supplementary Table S7), which was associated with the volume of the left and right amygdala (Supplementary Table S8). Mediation analysis showed the *ANKS1B* locus had an indirect effect on EverDrugs by the mediation of the left amygdala and right amygdala (Figure 2C). ANKS1B protein may be involved in the neural plasticity of the amygdala during drug use by affecting glutamatergic neurotransmission.^5^ Phenome‐wide association analysis based on the GWAS Atlas^6^ further validated the association of *ANKS1B*, *NRXN1* and *GTF2IRD1* with psychiatric traits (Supplementary Figure S7 and Supplementary Tables S9–S11).

We next explored the associated genes and pathways for the shared risk of HD and MD. *ANKS1B*, *GRM7*, *RBFOX1* and *CDH13* were shared by HD, MD and HD&MD (Supplementary Table S12). Enrichment analysis showed the HD‐ and MD‐related 22 unique genes were significantly enriched in brain‐related tissues, GO term ‘modulation of chemical synaptic transmission’ and drug abuse and neurodevelopmental disorder‐related diseases (Supplementary Figure S8). These associated genes and pathways provide new candidates and clues for understanding the genetic mechanism of drug dependences.

Additionally, polygenic associations with phenotypes of addiction‐related traits, risk behaviour, cognition and psychiatric disorders (Supplementary Table S13) were performed for HD, MD and AD. The three alcohol‐related traits were significantly associated with AD, but not with HD and MD (Figure 3A and Supplementary Table S14). Sexual and smoke behaviour risks were positively associated with the MD and HD&MD, but not with AD (Figure 3B and Supplementary Table S15). Cognition performance, education attainment and IQ were negatively associated with HD, MD and HD&MD but not with AD (Figure 3C and Supplementary Table S16). Our findings support that pre‐existing cognition disruption could increase the risk of illicit drug dependence.^7^ For the eight psychiatric disorders, only the PRS of ADHD was positively associated with HD, MD and HD&MD, but not with AD (Figure 3D and Supplementary Table S17). Mendelian randomisation analysis further highlighted the causal effect of ADHD on HD and HD&MD (Supplementary Table S18). Our previous study also demonstrated that ADHD‐relevant childhood behaviour was a risk factor for MA‐induced psychosis.^8^ First‐degree relatives of ADHD probands have an increased risk for drug dependence.^9^ This suggests that ADHD and drug dependence have shared genetic factors. Cluster analysis based on the standardised coefficient matrix from the PRS association results showed HD, MD and HD&MD were in one sub‐cluster and were different from AD, which further highlighted the closer polygenic correlations between HD and MD compared to AD.

**FIGURE 3:**
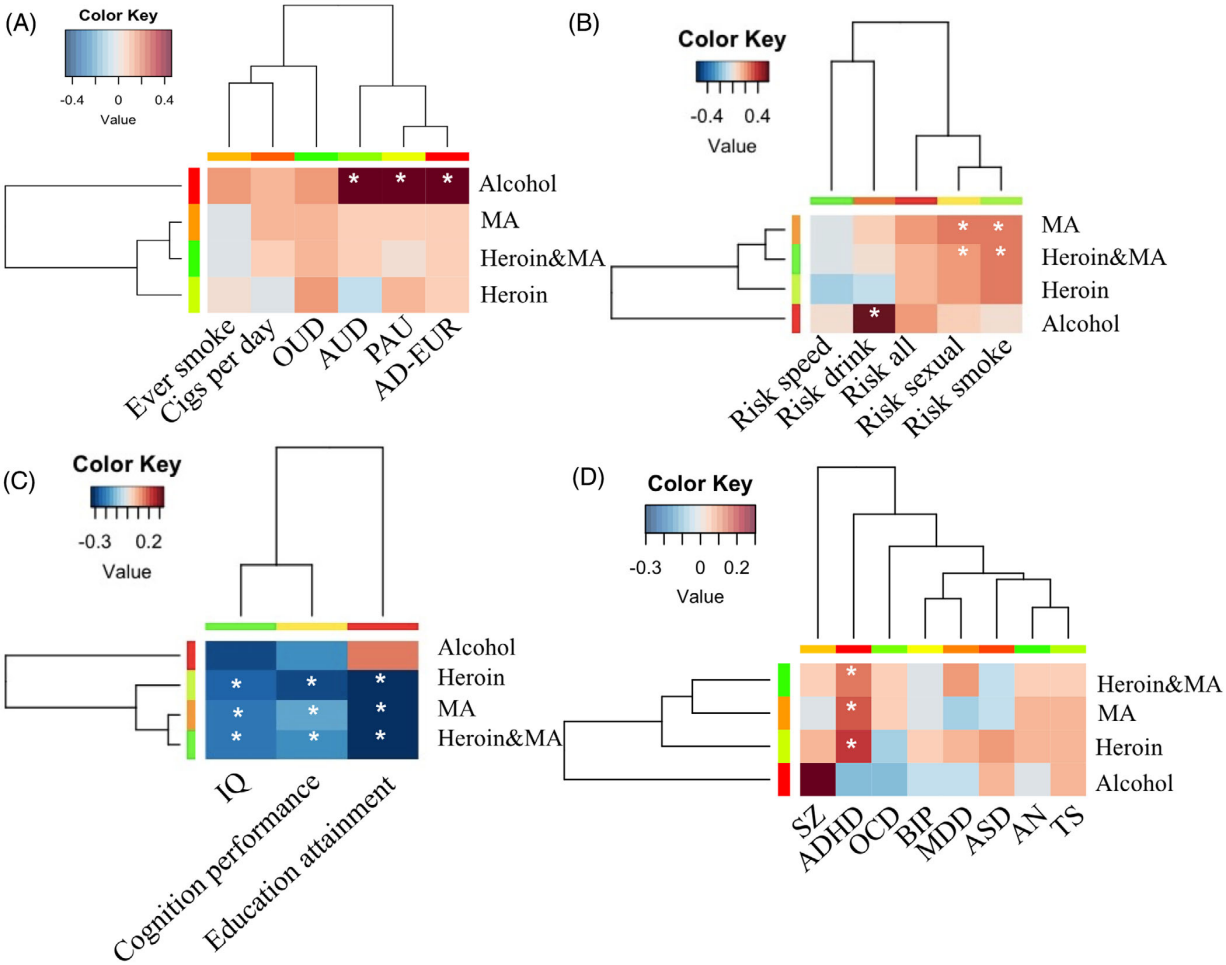
PRS analysis results for (A) six addiction‐related traits, (B) five risk behaviours, (C) three cognition traits and (D) eight psychiatric disorders with four SD traits. *denotes the permutation adjusted *p* was less than .05 in DS1 and replicated in DS2 (*p *< .05). Heatmap was constructed using the standardised beta coefficient. OUD: opioid use disorder, AUD: alcohol use disorder, PAU: problematic alcohol use, AD‐EUR: alcohol dependence of European population, SZ: schizophrenia, ADHD: attention‐deficit hyperactivity disorder, OCD: obsessive‐compulsive disorder, BIP: bipolar disorder, MDD: major depressive disorder, ASD: autism disorder, AN: anorexia nervosa, TS: Tourette syndrome.

In conclusion, at the genome‐wide loci, genes and polygenic levels, we identified significant genetic overlap between HD and MD, which distinguished with AD. Notably, the combined HD&MD GWAS identified three common risk loci, located on *ANSK1B*, *NRXN1* and *GTF2IRD1* genes. At the polygenic level, HD and MD were significantly associated with cognition deficiency and ADHD, which distinguished them from AD. Our results would help with the fine‐mapping of the common and unique genetic mechanisms underlying drug dependences and alcohol dependence and may provide clues for their prevention.

## CONFLICT OF INTEREST

The authors declare no competing interest.

## Supporting information

Supporting Information
