# Supplementary material for: Genome‐wide association meta‐analyses identify novel genetic risk loci and polygenic phenotype associations for heroin, methamphetamine and alcohol dependences
Source: Clin Transl Med. 2022 Jan 24;12(1):e659. doi: 10.1002/ctm2.659 (PMC8787099; doi:10.1002/ctm2.659)
Supplement: Supplementary file 1 — Supporting Information [file CTM2-12-e659-s001.pdf]

Supplementary Materials

Genome-wide association meta-analyses identify novel genetic risk loci and polygenic phenotype associations for heroin, methamphetamine, and alcohol dependences

Su-Hua Chang[1], Yan Sun[2,3], Fan Wang[4], Xiang-Wen Chang[2], Ying-Jian Zhang[1], Tian-Ye Jia[5,6], Hong-Qiang Sun[1], Wei-Hua Yue[1], Ping Wu[2,3], Lin Lu[1,2,*], Jie Shi[2,3,7,8,*]

[1] Peking University Sixth Hospital, Peking University Institute of Mental Health, NHC Key Laboratory of Mental Health (Peking University), National Clinical Research Center for Mental Disorders (Peking University Sixth Hospital), Chinese Academy of Medical Sciences Research Unit (No.2018RU006), Peking University, Beijing, China

[2] National Institute on Drug Dependence, Peking University, Beijing, China

[3] Beijing Key Laboratory on Drug Dependence Research, Peking University, Beijing, China

[4] Beijing HuiLongGuan Hospital, Peking University HuiLongGuan Clinical Medical School, Beijing, China

[5] Social, Genetic and Developmental Psychiatry Centre, Institute of Psychiatry, Psychology & Neuroscience, King's College London, De Crespigny Park, London, United Kingdom

[6] Institute of Science and Technology for Brain-Inspired Intelligence, Ministry of Education-Key Laboratory of Computational Neuroscience and Brain-Inspired Intelligence and Research and Research Institute of Intelligent Complex Systems, Fudan University, Shanghai, China

[7] The State Key Laboratory of Natural and Biomimetic Drugs, Peking University China

[8] The Key Laboratory for Neuroscience of the Ministry of Education and Health, Peking University, China

Su-Hua Chang and Yan Sun contributed equally to this work.

[2] To whom correspondence may be addressed. shijie@bjmu.edu.cn or linlu@bjmu.edu.cn

Supplementary Methods

1. Study participants

Two datasets of substance-specific dependence patients, with only one dependence for either heroin, methamphetamine, or alcohol (HD, MD, AD), from the Han Chinese population, were used in the GWAS meta-analysis. The first dataset (DS1) included 1088 HD, 1773 MD, 571 AD and 3067 shared controls, which has been included in our previous GWAS study(1). The second dataset (DS2), which was newly generated, included 1003 HD, 719 MD, 228 AD and 1128 shared controls.

(1) Study participants for dataset 1 (DS1, genotyped using GSA chip)

1) Alcohol dependence patients: Hospitalized alcohol dependence patients were recruited from twelve psychiatric hospitals in northern China (Beijing, Inner Mongolia Autonomous Region, Shandong, Tianjin, Jilin, Liaoning, and Heilongjiang provinces) from Sept. 2009 to Nov. 2011. All enrolled patients sought treatment for alcohol dependence (AD) and were clinically ascertained as AD based on DSM-IV by experienced psychiatrists. Patients had not used any other addictive substances except for nicotine based on self-reports and medical record. All study subjects had no history of other major psychiatric disorders and medical conditions based on self-reports.

2) Heroin dependence patients: Heroin dependence patients of dataset 1 were from five drug addiction treatment centers in China's Guangdong and Hubei provinces (Yichang, Sanshui, Shenzhen, and Huizhou) and were enrolled from Jan. 2011 to Mar. 2014. According to the self-reported questionnaires and medical records, heroin was their primary drug of abuse. Confirmatory urine tests were positive for heroin at the time of treatment. Individuals met the DSM-IV criteria for heroin dependence as determined by trained interviewers. Study patients had no history of polysubstance dependence (continuous use of other opioid drugs for no more than one month and use of other kinds of addictive drugs for no more than three times within the past three years) except for nicotine dependence. Alcohol dependence was also excluded by selecting patients with a score < 4 in the Michigan Alcoholism Screening Test. All study

subjects had no history of other major psychiatric disorders and medical conditions based on self-reports.

3) Methamphetamine dependence patients: MA dependence patients were recruited from three addiction treatment centers in China's Guangdong province (Dongguan, Shenzhen, and Sanshui) from Dec. 2016 to Mar. 2017. Using self-reported questionnaires and government records, MA was their primary drug of abuse, and urine tests confirmed MA use at the time of treatment. Patients met the DSM-V criteria for methamphetamine dependence and were determined by trained interviewers. Patients did not use other non-amphetamine-type stimulants (e.g., opioids and cocaine) for more than three times per year within the past three years, except for nicotine. Alcohol dependence was also excluded by selecting patients with a score < 4 in the Michigan Alcoholism Screening Test. All study subjects had no history of other major psychiatric disorders and major medical conditions based on self-reports.

4) Healthy controls: Healthy controls (HCs) were recruited from local communities through advertisements and community centers. All HCs did not have a history of substance dependence (except nicotine), psychiatric disorders, or any current major medical conditions (e.g., cardiovascular, endocrinological, oncological, or autoimmune diseases) based on self-report questionnaires.

(2) Study participants for dataset 2 (DS2, genotyped using ASA chip)

1) Heroin dependence patients and methamphetamine dependence patients: In-patients who were dependent on just heroin or methamphetamine were from Sanshui addiction treatment hospital in Guangdong province of China. They were enrolled in Mar. 2018. The inclusion criteria (except for updating the DSM-V criteria for heroin dependence) and exclusion criteria were consistent with DS1.

2) Alcohol dependence patients: AD inpatients were recruited from the Peking University Sixth Hospital and four other psychiatric hospitals from Anhui, Shangdong, and Henan provinces, China in 2018. The inclusion (except for updating the DSM-V criteria for alcohol dependence) and exclusion criteria were consistent with DS1.

3) Healthy controls: 101 HCs were recruited from local communities, others were first-year medical college students (age > 16) from 8 universities located in several south China provinces (Hubei, Guizhou, Sichuan) and north China provinces (Hebei, Heilongjiang, Shandong, and Inner Mongolia). All students were of Han ethnicity. The recruitment method and inclusion criteria were the same as the DS1 controls.

All study participants were informed of the entire procedure and potential risks before being requested to sign a written informed consent form. Participants received monetary compensation for participating in the study.

In addition to diagnostic information, related addiction characteristics, including cravings, dose per time (g), and usage frequency per day within the past year were also collected for HD and MD patients. For drug craving, we used the visual analog scale (VAS, 0-10) to assess the self-reported average craving degree across the past year. The self-reported dose per time (g) and usage frequency per day of the most recent before treatment were recorded for HD and MD respectively. The study was approved by the Institutional Review Board of Peking University Health Science Center.

2. Quality control of the GWAS data

The genotypes of the samples in DS1 and DS2 were obtained using the Infinium Global Screening Array-24 v1.0 BeadChip (GSA) and Infinium Asian Screening Array-24 v1.0 BeadChip (ASA), respectively. The same quality control and imputation pipeline were used for each case-control pair of SD traits for each dataset separately (AD, HD, MD, HD&MD) using PLINK version 1.9(2). SNPs with call rates < 95%, minor allele frequency (MAF) < 0.01 or Hardy-Weinberg (H-W) equilibrium test P<1.0e-6 were removed. Data on individuals lacking information on age or gender, call rate < 95%, autosomal heterozygosity > 5 s.d. away from the mean, or with lower call rates from a pair of individuals with proportion identity by descent (IBD) PI_HAT ⩾ 0.185 were removed. The number of samples after quality control for the two GWAS datasets are shown in Table S1. Genotype imputation was performed using the prephasing/imputation stepwise approach implemented in IMPUTE2(3) and SHAPEIT(4). The

imputation reference set consisted of 2,186 phased haplotypes from the full 1000 Genomes Project Integrated Phase 1 Release (March 2012)(5). The number of SNPs after QC, after imputation and overlapped SNPs for meta-analysis for each data set are shown in Table S2. Principal component analysis (PCA) was performed for each case-control GWAS using SNPs with low linkage disequilibrium (LD) (MAF > 0.35 and $r^2 < 0.05$) and absent from the 5 long-range LD regions(6) using the EIGENSOFT 4.2 software(7, 8). The PCA plot for the first two PCs in the two data sets is shown in Figure S1. The top 10 PCs were used as covariates for the association tests. SNPs with info > 0.6 and minor allele frequency > 0.01 were used for the association test.

3. Genome-wide association analysis (GWAS) and meta-analysis

GWAS analysis for each case-control SD trait (HD, MD, AD, HD&MD) was performed for DS1 and DS2 separately. For GWAS of HD&MD, the samples with HD or MD were combined as cases for each dataset and then compared with controls to generate the GWAS summary data for DS1 and DS2 separately. The association test was performed using SNPTEST. Age, gender, and the 10 PCs from population stratification analyses were used as covariates. Meta-analyses for the two datasets were performed using the METAL STDERR model(9). Only SNPs with the same direction in the two datasets were retained in the meta-analysis results. SNPs with P_{meta}< 5e-8 and P<0.05 in both datasets were considered significant. SNP heritability and genetic correlation between addiction traits were calculated using LD score regression (LDSC)(10) based on the meta-analysis GWAS summary data.

4. Functional mapping and annotation

We used the Functional Mapping and Annotation of genetic associations (FUMA) v1.3.6 (https://fuma.ctglab.nl) to annotate GWAS data and obtain a functional characterization of risk loci (11). The SNP2Gene module was used to define independent genomic risk loci and variants in LD with lead SNPs (r^2>0.6, calculated using ASN 1KGP reference genotypes). Functional consequences for SNPs were obtained by mapping the SNPs on their chromosomal position and reference alleles to databases containing known functional annotations, including ANNOVAR, Combined Annotation Dependent Depletion (CADD), RegulomeDB (RDB), and

chromatin states (only brain tissues/cell types were selected). Gene expression data for all tissues and eQTL mapping for the 13 brain-related tissues were performed using the data from the GTEx v8 database(12).

5. Association analysis with related brain volume

We used the Human Connectome Project (HCP) data to explore the association of the significant loci with illicit drug use and related brain region volumes. The data were drawn from 1206 participants (all provided full informed consent) from the HCP database (March 2017 public data release) from the Washington University-University of Minnesota (WU-Minn HCP) Consortium. The research procedures and ethical guidelines were approved by Washington University's institutional review board. The drug-related trait, EverDrugs, which was defined as 1 if the participant had ever used illicit drug, otherwise 0, was used as the main phenotype.

The high-resolution T1-weighted (T1w) and T2-weighted (T2w) structural images for the participants were acquired on a 3T Siemens Skyra scanner (Siemens AG, Erlanger, Germany) with a 32-channel head coil at a resolution of 0.7 mm isotropic (field of view [FOV] = 224 × 224, matrix = 320 × 320, 256 sagittal slices, repetition time [TR] of T1w = 2400 ms, TR of T2w = 3200 ms, echo time [TE] of T1w = 2.14 ms, TE of T2w = 565 ms). The brain volume data used in this study were derived from the HCP preprocessed pipeline using FreeSurfer and included total intra-cranial volume, subcortical segmentation volume for the amygdala, thalamus, pallidum, hippocampus, putamen, caudate, and accumbens of each hemisphere (13, 14).

For genotype data, we tried to extract all SNPs with LD $r^2>0.6$ with the lead SNP in the GWAS meta-analysis result of HD&MD using the GWAS genotype data for HCP samples, which was requested from dbGaP using accession number phs001364. Four (rs10860447, rs2202037, rs1912861, rs78362061), two (rs6750490, rs1363047) and one (rs76965632) SNPs were extracted for the ANKS1B locus, NRXN1 locus and GTF2IRD1 locus respectively.

In total, 1050 samples had all data for the association analysis, including EverDrugs, genotype data for the significant SNPs, brain region volumes and related covariates. The

association analysis for the SNPs with EverDrugs was analyzed using logistic regression models with age, gender, race, education, and BMI as covariates. In addition, association analyses for brain volume with EverDrugs, and SNP with brain volume, the total intra-cranial volume was included as a covariate as well. Brain mediation analysis was performed using process in SPSS. Since we had expected directionality of the correlations, one-tailed test P-values were used.

6. Gene-based and gene-set analysis using MAGMA

Gene-level association analysis with SNP-wise mean model and gene-set association analysis was performed using MAGMA in FUMA(11, 15). Input SNPs were mapped to 18034 protein-coding genes. Genome-wide significance for genes were defined at p = 0.05/18034 = 2.773e-6. For gene-set association analysis, 10678 gene sets (curated gene sets: 4761, GO terms: 5917) from MsigDB v6.2 were used. Bonferroni correction was performed for all the tested gene sets. Tissue enrichment analyses for the drug dependence-related genes were performed using GENE2FUC in FUMA(11). Functional enrichment for Gene Ontology (GO) terms and disease-gene network database DisGeNET(16), which integrates human gene-disease associations from various databases for a large number of Mendelian and complex diseases, were performed using Metascape(17) to show the function of the drug dependence-related genes.

7. GWAS summary data for polygenic risk score (PRS) analysis

We used GWAS summary data from four groups of phenotypes for PRS analysis. The GWAS data-related reference, sample size, and number of SNPs are shown in Table S13.

(1) Six addiction-related traits, including 1) alcohol dependence, relied on the "EU GWAS of unrelated genotyped individuals (excluding summary statistic cohorts)", 2) ever smoke and 3) cigs per day were downloaded from the Tobacco and Genetics Consortium(18). 4) problematic alcohol use (PAU)(19), from the GWAS meta-analysis for the samples from MVP (Million Veteran Program), PGC and UK Biobank. 5) alcohol use disorder (AUD)(20), from the GWAS meta-analysis for the samples from MVP and PGC. 6) opioid use disorder (OUD)(21), which was from the trans-ancestry analysis using the samples of MVP and Yale-Penn and Study of

Addiction: Genetics and Environment samples. The GWAS summary data for PAU, AUD and OUD were downloaded from dbGaP using accession number phs001672.

(2) Risk tolerance, including 1) general risk tolerance, 2) risk speeding (automobile speeding propensity), 3) risk drinking (drinks per week), 4) risk smoking (ever smoker) and 5) risk sex (number of sexual partners). The all-SNP summary data were obtained from the literature(22).

(3) Three cognition-related traits, including 1) educational attainment and 2) cognitive performance were obtained from the Social Science Genetic Association Consortium (SSGAC) (https://www.thessgac.org/data)(23). Note, the GWAS summary data for educational attainment excluded 23andMe. 3) intelligence, was obtained from a meta-analysis of 269,867 individuals(24).

(4) Eight psychiatric disorders, including 1) attention deficit hyperactivity disorder (ADHD), 2) major depressive disorder (MDD), 3) bipolar disorder (BIP), 4) schizophrenia (SZ), 5) autism disorder (ASD), 6) anorexia nervosa (AN), 7) obsessive-compulsive disorder (OCD) and 8) Tourette syndrome (TS) were used for PRS analyses. GWAS summary data for ADHD, BIP, ASD, AN, OCD, and TS were downloaded from the PGC website (http://www.med.unc.edu/pgc/results-and-downloads). The GWAS summary data for SZ was from the latest SZ GWAS, which combined PGC data and Chinese individuals (downloaded from http://analysis.bio-x.cn/gwas/). The GWAS summary data for MDD was derived from the meta-analysis that integrated MVP samples and UK Biobank data(25).

8. Polygenic risk score analysis

First, pairwise PRS associations of HD, MD, and AD across DS1 and DS2 (using DS1 GWAS summary data to calculate PRS of DS2 (or vice versa)) were analyzed to further determine their genetic correlation. PRSice-2 was used to calculate PRS(26). Before generating the scores, clumping was used to obtain SNPs in linkage equilibrium with an $r^2<0.1$ within a 250 bp window. PRS was then generated using SNPs at *P*-value thresholds from 0 to 0.5 with a stepwise increase of 0.00005. Associations between the polygenic profile and SD traits (AD, HD, MD, HD&MD) were examined using multiple logistic regression models after adjusting

for age, sex, and the top 10 PCs. Other parameters used the default value. The *P*-value threshold with the biggest Nagelkerke's r^2 (variance explained by PRS) was considered as the best-fit threshold. The *P*-value was adjusted by using 10,000 label-swapping permutations. For the PRS analysis using DS1/DS2 as the discovery and DS2/DS1 as the target, $P_{empirical}<0.05$ was defined as significant.

Afterward, PRS analyses using related phenotypes (Table S13) were performed to compare the effect of PRS from different related phenotypes on SD traits. The distribution of the polygenic risk score (PRS) for addiction-related phenotypes (ADHD, Cognition Performance, Risk Sexual as examples) in the two datasets (DS1 and DS2) showed the PRS was normally distributed. This suggested the PRS could be used in DS1 and DS2 (Figure S9). We performed normalization for the PRS using the best *P*-value threshold and further performed regression modeling using the same covariates in R to obtain the standardized beta coefficient. PRS analyses were initially performed for DS1, then the association of the PRS at the same best *P*-value threshold for DS1 was used for DS2. If the PRS result for DS1 was significant ($P_{empirical}<0.05$) and the PRS was also significant ($P<0.05$) in DS2, we considered the results to be replicated. The R package metafor was used to obtain the estimate and standard error for the meta-analysis results. Cluster analyses for both SD traits and related phenotypes were additionally performed based on the coefficient matrix from the meta-analysis results using heatmap.2 R package to generate the dendrogram.

9. Causal relationship analysis for the significant PRS associations using two-sample Mendelian randomization

We performed two-sample Mendelian randomization analyses (MR)(27) for all significant polygenic correlations with phenotypes from PRS analysis using the R package TwoSampleMR(28). Inverse-variance-weighted (IVW) regression(29) was used to perform the MR test of a causal effect. The MR Egger intercept test was used to test directional horizontal pleiotropy and the IVW regression test for variant heterogeneity. Additional sensitivity tests were performed using the MR Egger test and weighted median test.

Using SNPs with *P*<5e-8 in the PGC2 ADHD GWAS as the instrument variable, ADHD had a causal effect on HD (IVW test *P*=0.014) without evidence of global horizontal pleiotropy (*P*=0.947) and heterogeneity (*P*=0.917) (Table S18). The causal effect of ADHD still existed for both HD and HD&MD when the instrument variable used ADHD SNPs with *P*<e-6. Sensitivity analyses showed both MR Egger and weighted median methods had effects in the same direction as the IVW test. This demonstrated the results were consistent among the three methods.

Causal analysis showed lower EA, CP and IQ had causal effects on HD and MD. The causal effect of risk drink on AD, risk sexual, and smoke on HD&MD were also significant. However, heterogeneity tests were significant, and some inconsistencies were present for the sensitivity test (Table S18). The heterogeneity or inconsistent effect among the different methods suggests these causal relationships may be affected by other confounders and need further study.

10. Limitations of this study

Our findings delineated similar genetic susceptibility between HD and MD at genome-wide loci, gene, and polygenic levels, and distinguished them from AD. However, there were several limitations to our study. First was the limited statistical power. Although our sample size was the largest SD study using an Asia population, and we found several novel loci for drug dependence, it may not allow for comprehensively describing the genetic architecture of addictions. The association result identified in this study needs to be replicated in an independent cohort. In addition, since we have no smoking data for controls, we didn't add smoking as covariate in the GWAS analysis, which may lead bias on the result. Furthermore, for polygenic analysis, since there was no available GWAS data or the sample cohort was small for the Chinese population, the GWAS summary data of phenotypes were derived from European cohorts. This may lead to bias and hence reduce the prediction accuracy due to ethnic differences(30). We intent to update our findings when additional GWAS data is available from Chinese ethnic populations.

Figure S1. PCA plot for all samples in DS1 (A) and DS2 (B).

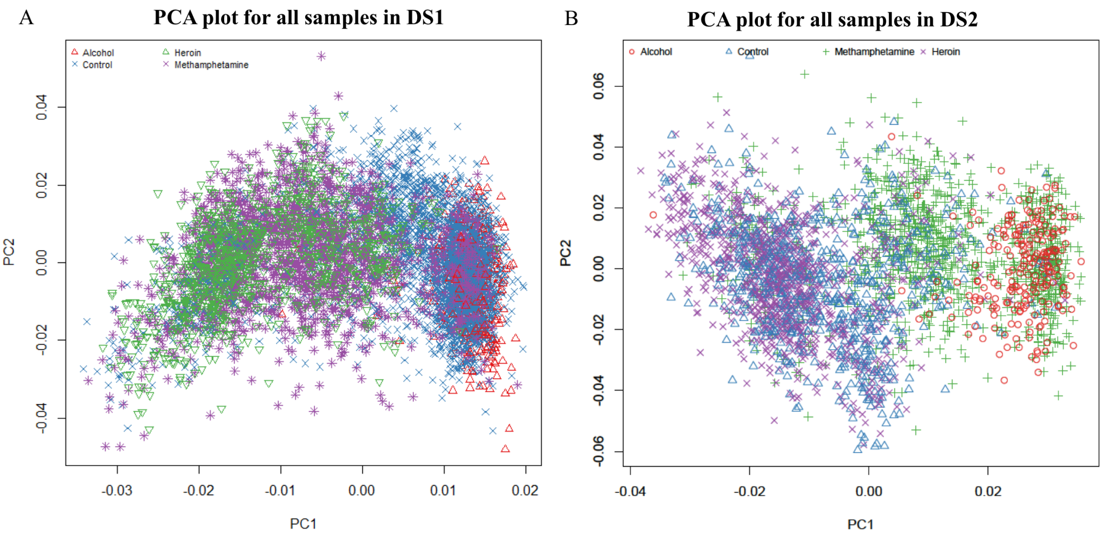

Figure S2. Q-Q plot for each GWAS in DS1 and DS2. Lambda GC, LDSC intercept and ratio for each GWAS are marked for each plot.

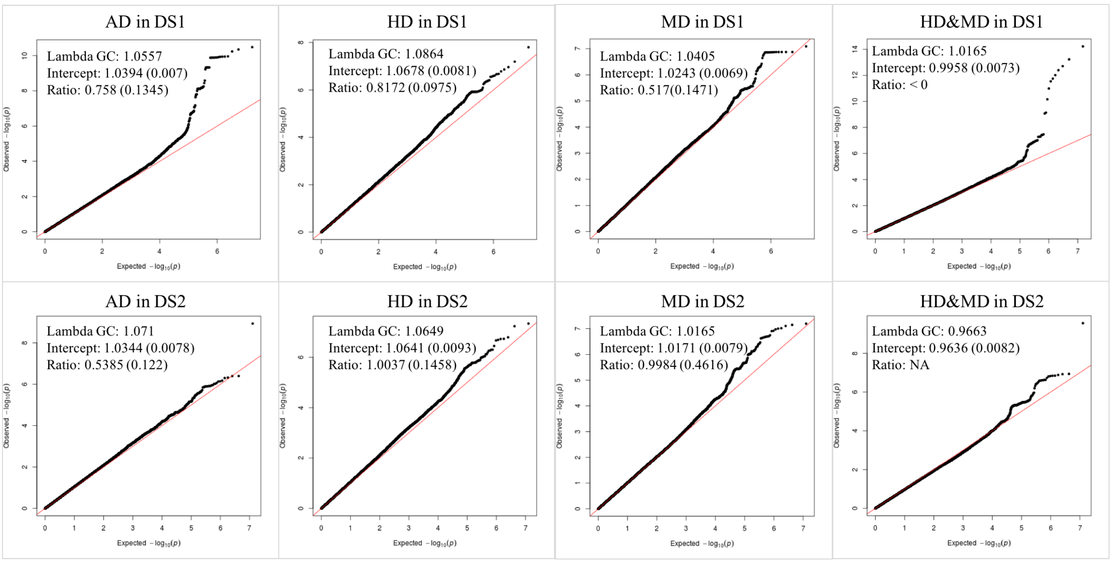

Figure S3. (a) Manhattan plot of the meta-analysis for alcohol dependence (AD). Two significant loci at *ADH1B* in chr4 and *ALDH2* in chr12 were found. Regional plots for the two significant loci are in (b) and (c).

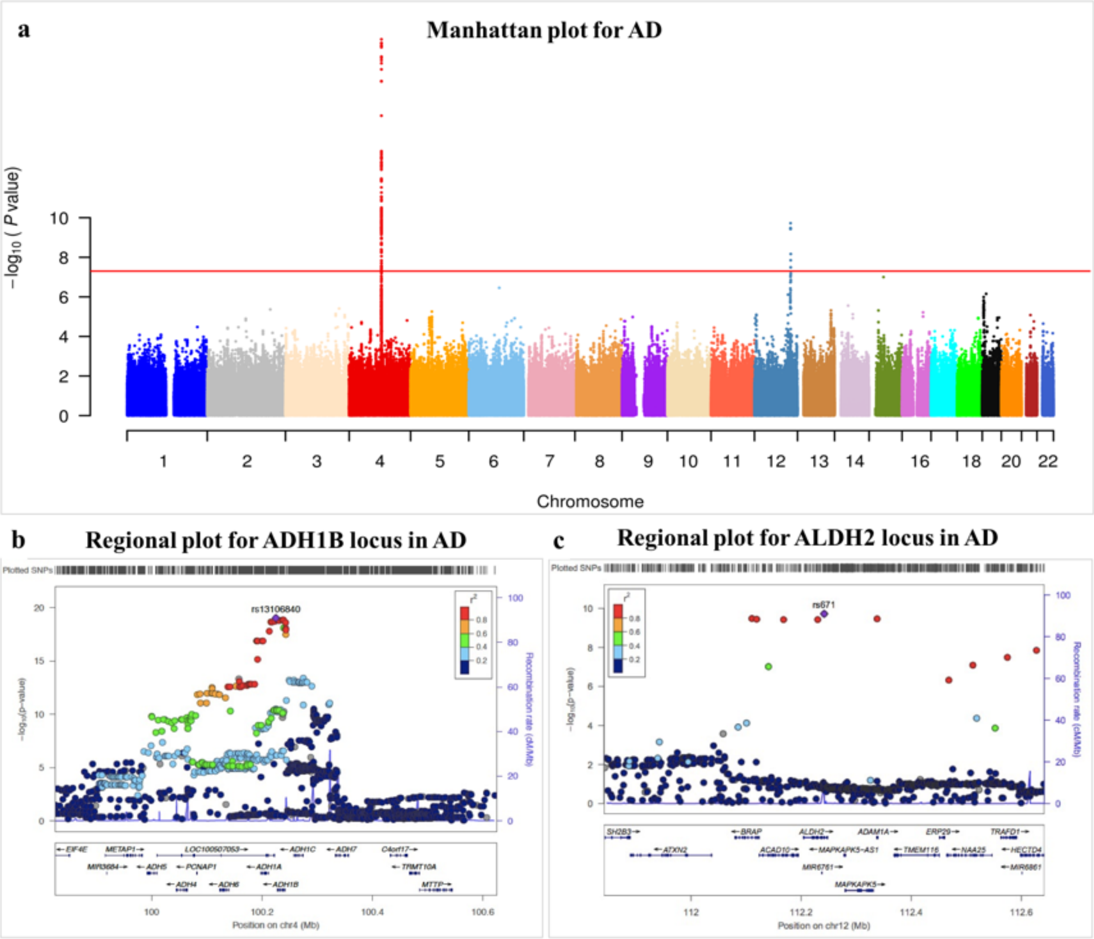

Figure S4. Functional annotation for the three significant loci of HD&MD. (a) chr12 *ANKS1B* locus significant for HD, MD, and HD&MD; (b) chr2 *NRXN1* locus significant for HD&MD, (c) chr7 *GTF2IRD1* locus significant for HD&MD. Figures were generated using FUMA "regional plot with annotation". The chromatin state of neuronal cell lines/tissues are shown, and included E007, E009, E010, E053, E054, E067, E068, E069, E070, E071, E072, E073, E074, E081, E082, and E125 in Roadmap Epigenomics.

(a) chr12 *ANKS1B* locus significant for HD, MD, and HD&MD

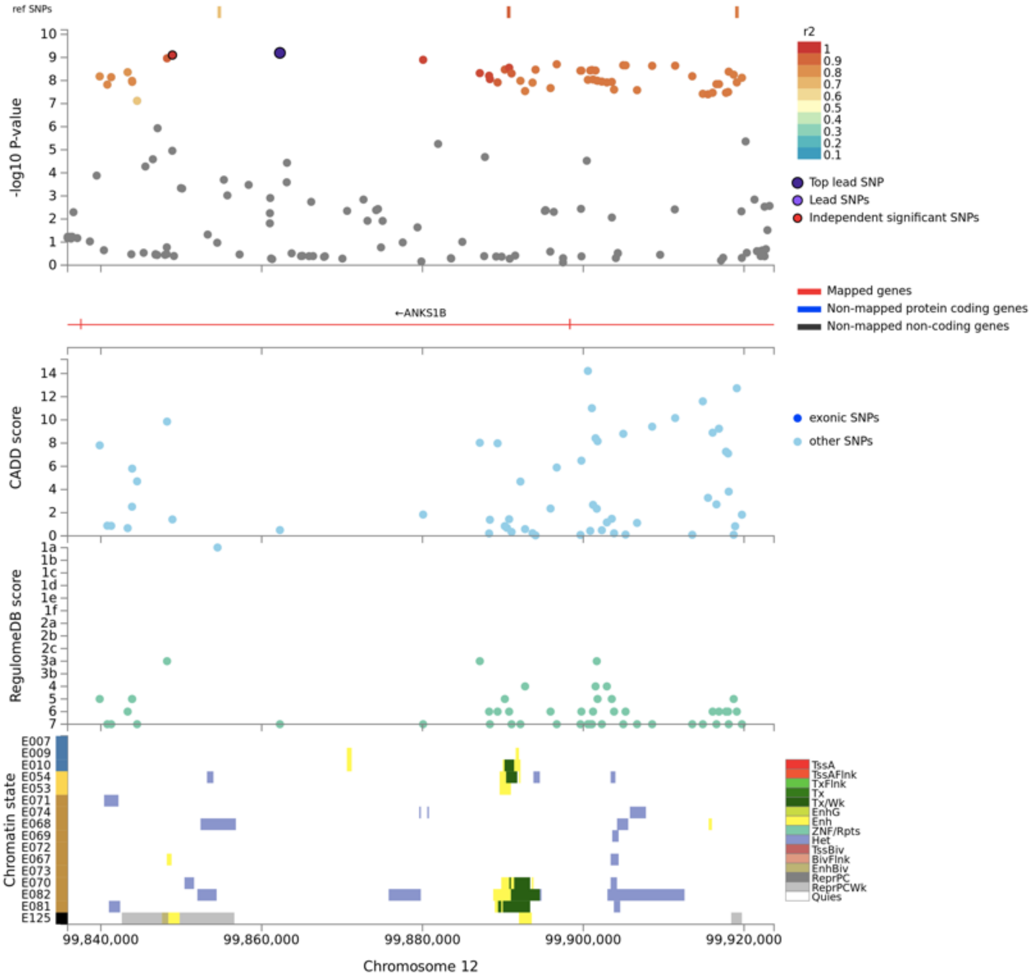

(b) chr2 *NRXN1* locus significant for HD&MD

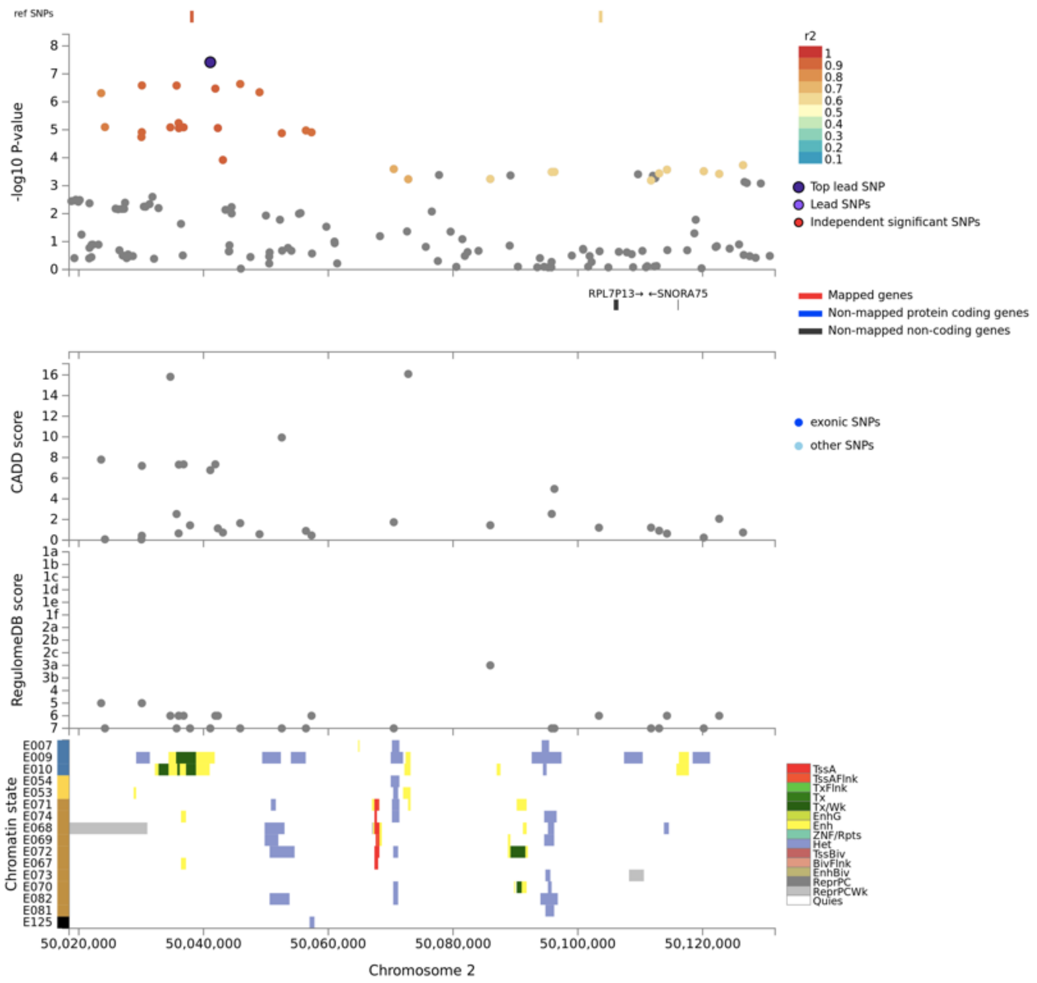

(c) chr7 *GTF2IRD1* locus significant for HD&MD

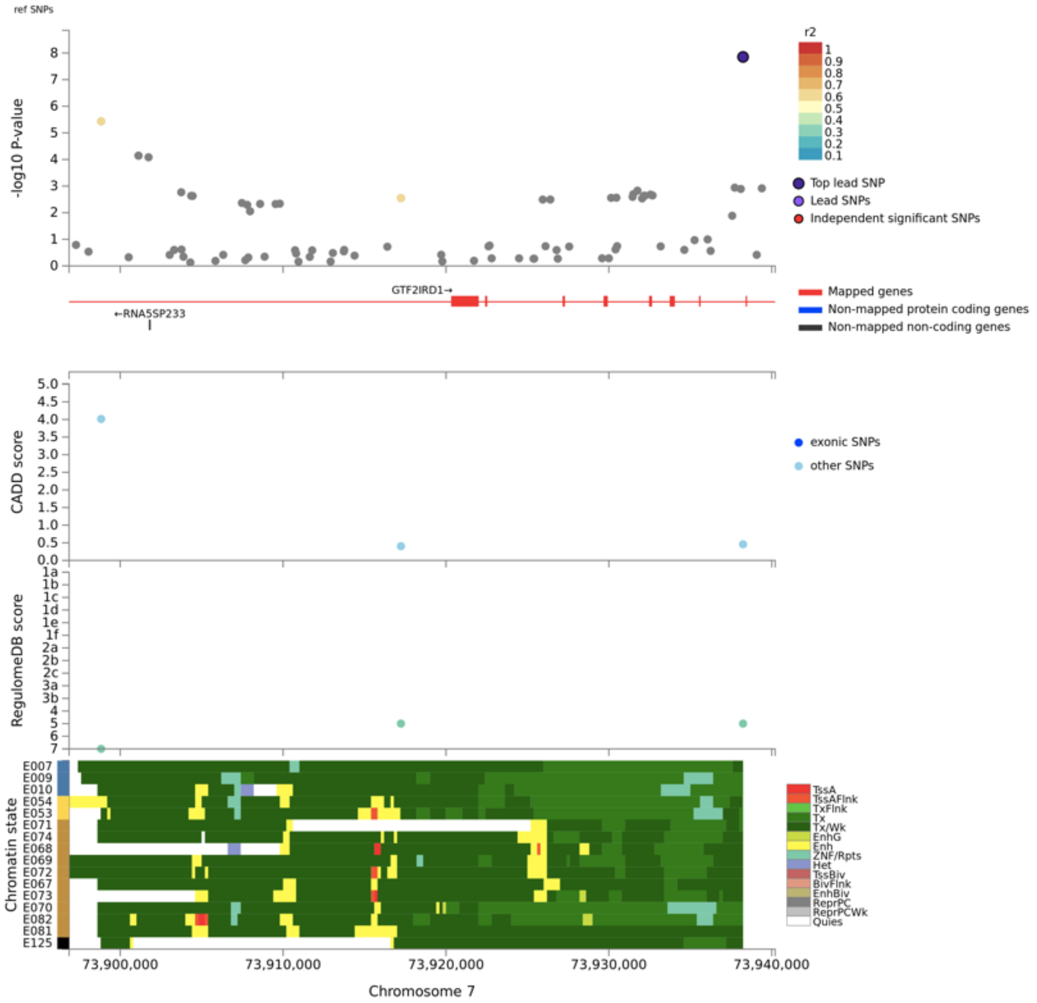

Figure S5. Expression profiles of the three genes located in the significant loci for HD&MD in different tissues derived from GTEx data. (a) *ANKS1B*, (b) *NRXN1*, (c) *GTF2IRD1*.

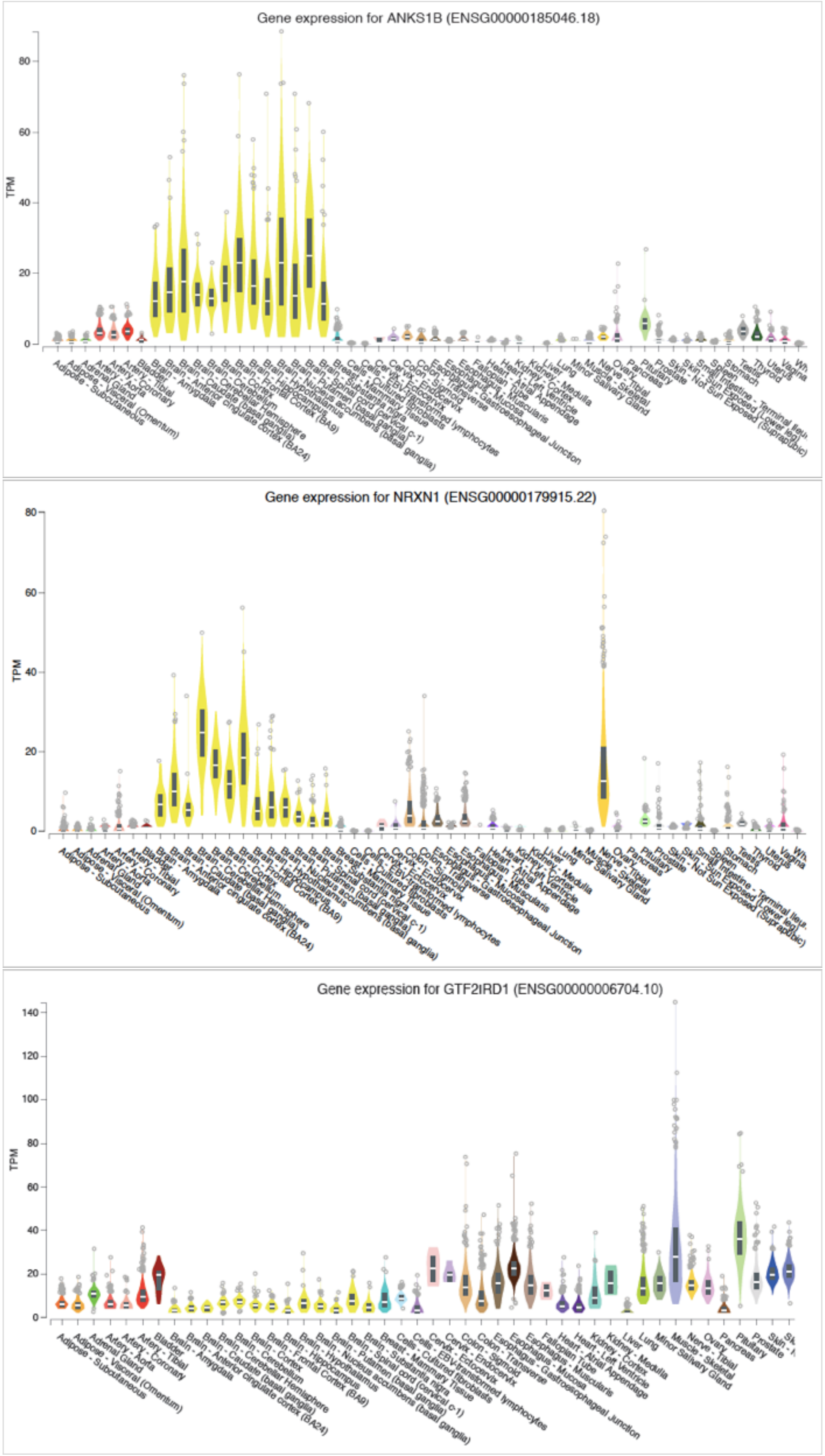

Figure S6. Comparison of the five significant loci for each type of substance dependence trait to demonstrate the genomic relationships of different substance dependences.

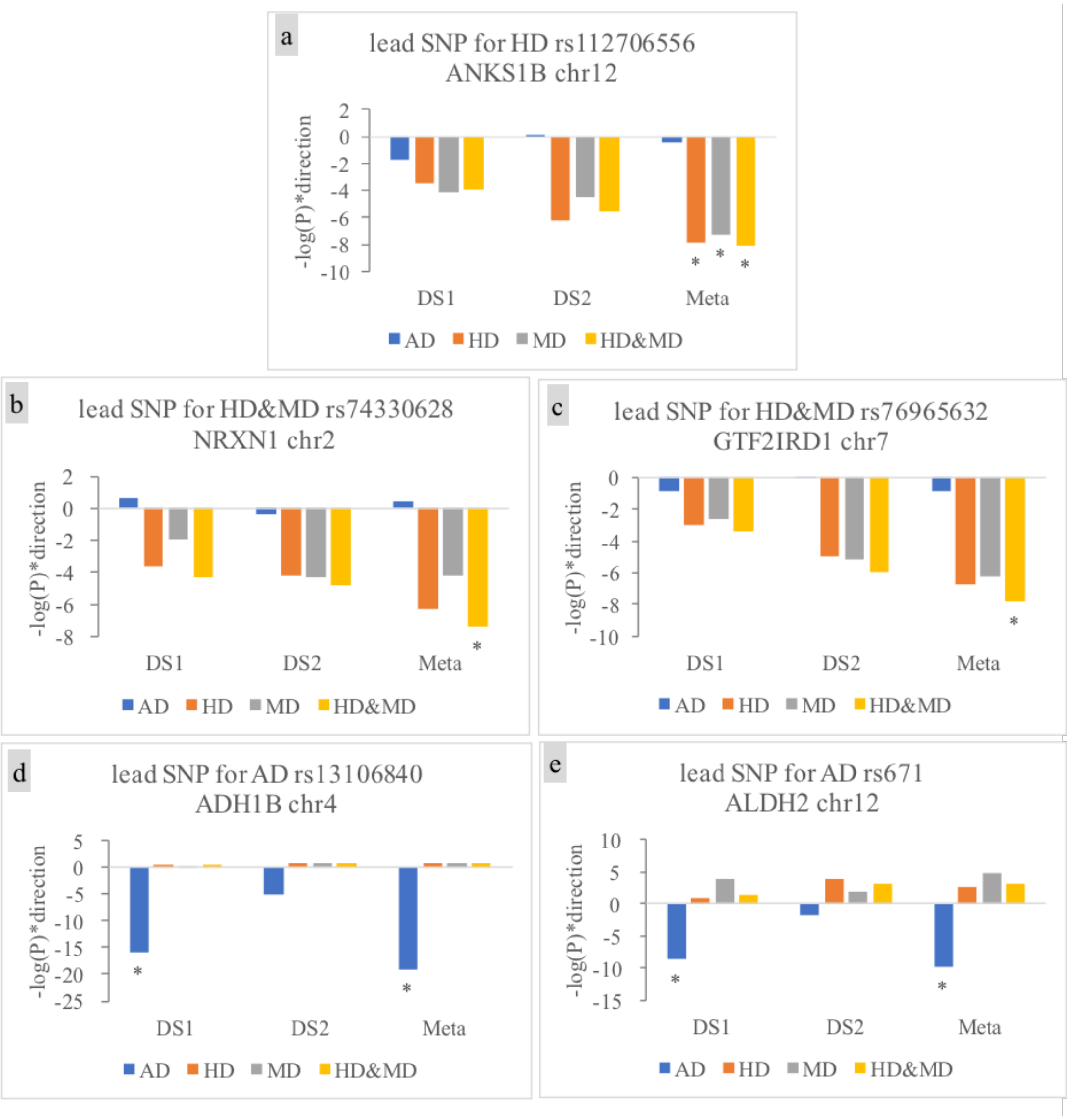

Figure S7. PheWAS analysis results for the three genes: (A) *ANKS1B*, (B) *NRXN1*, and (C) *GTF2IRD1*. Plots were generated using https://atlas.ctglab.nl/PheWAS by inputting the gene name. *ANKS1B* and *NRXN1* were mainly associated with metabolic and psychiatric traits. *GTP2IRD1* was mainly associated with psychiatric traits. Number of GWASs considered: 4756 (including GWASs in which the searched SNP or gene was not tested, Bonferroni corrected P-value: 1.05e-5). Red lines denote the threshold of P-value<1.05e-5.

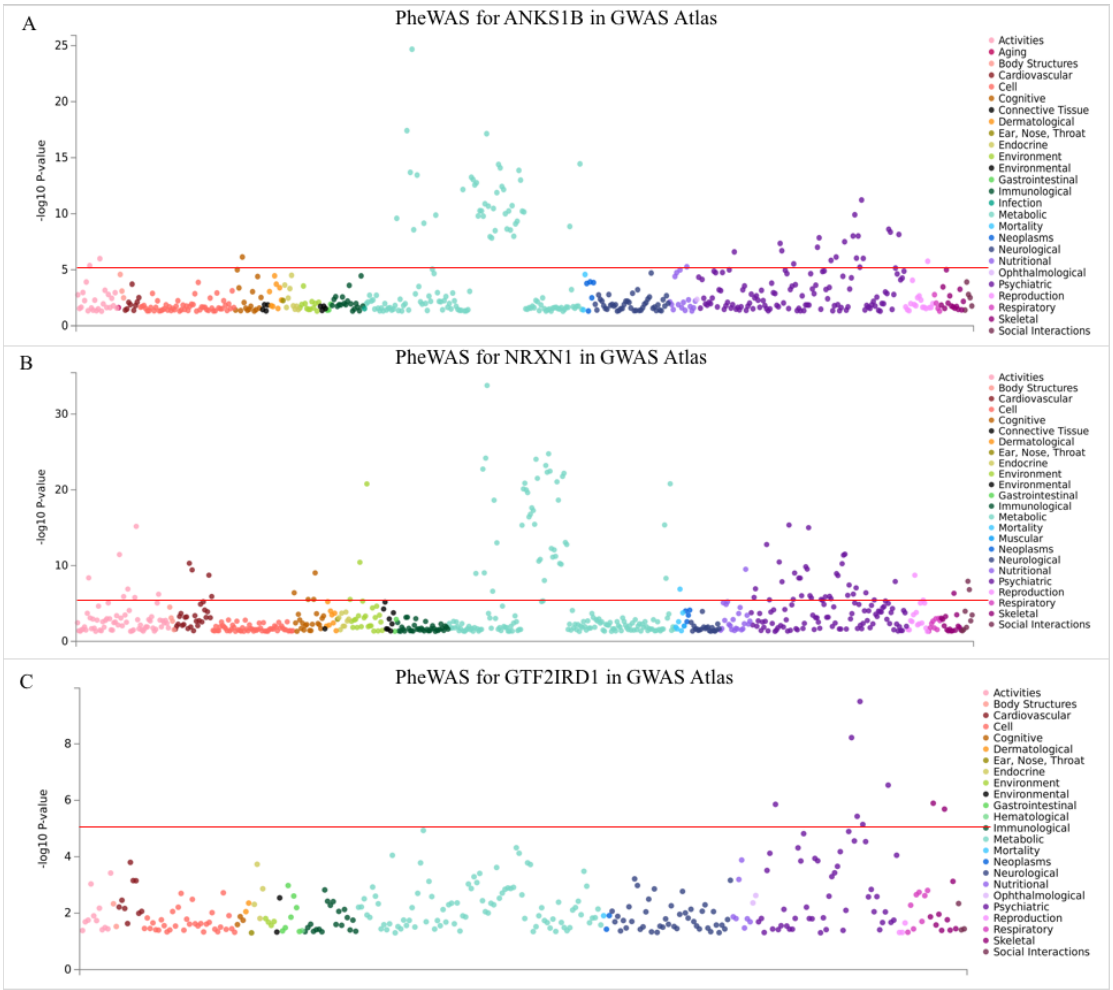

Figure S8. Enrichment analysis for the 22 significant genes for drug dependence (HD, MD, HD&MD). a) tissue enrichment analysis results using FUMA; b) functional enrichment analysis in GO using Metascape. c) enrichment analysis in DisGeNET (one of the largest available collections of genes and variants involved in human diseases(16)) using Metascape.

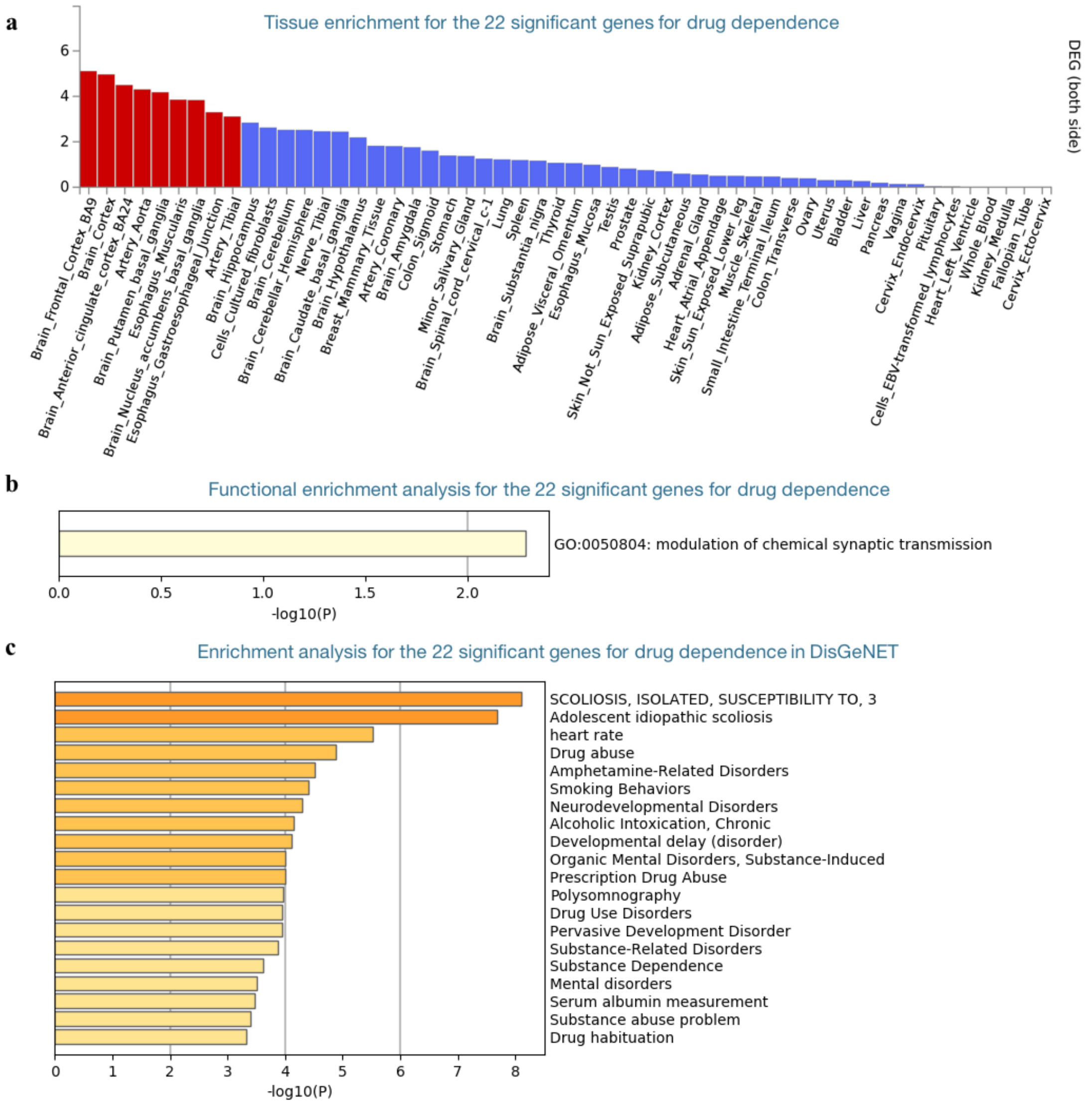

Figure S9. Distribution of the polygenic risk score (PRS) for addiction-related phenotypes (ADHD, Cognition Performance, Risk Sexual as examples) in the two datasets (DS1 and DS2).

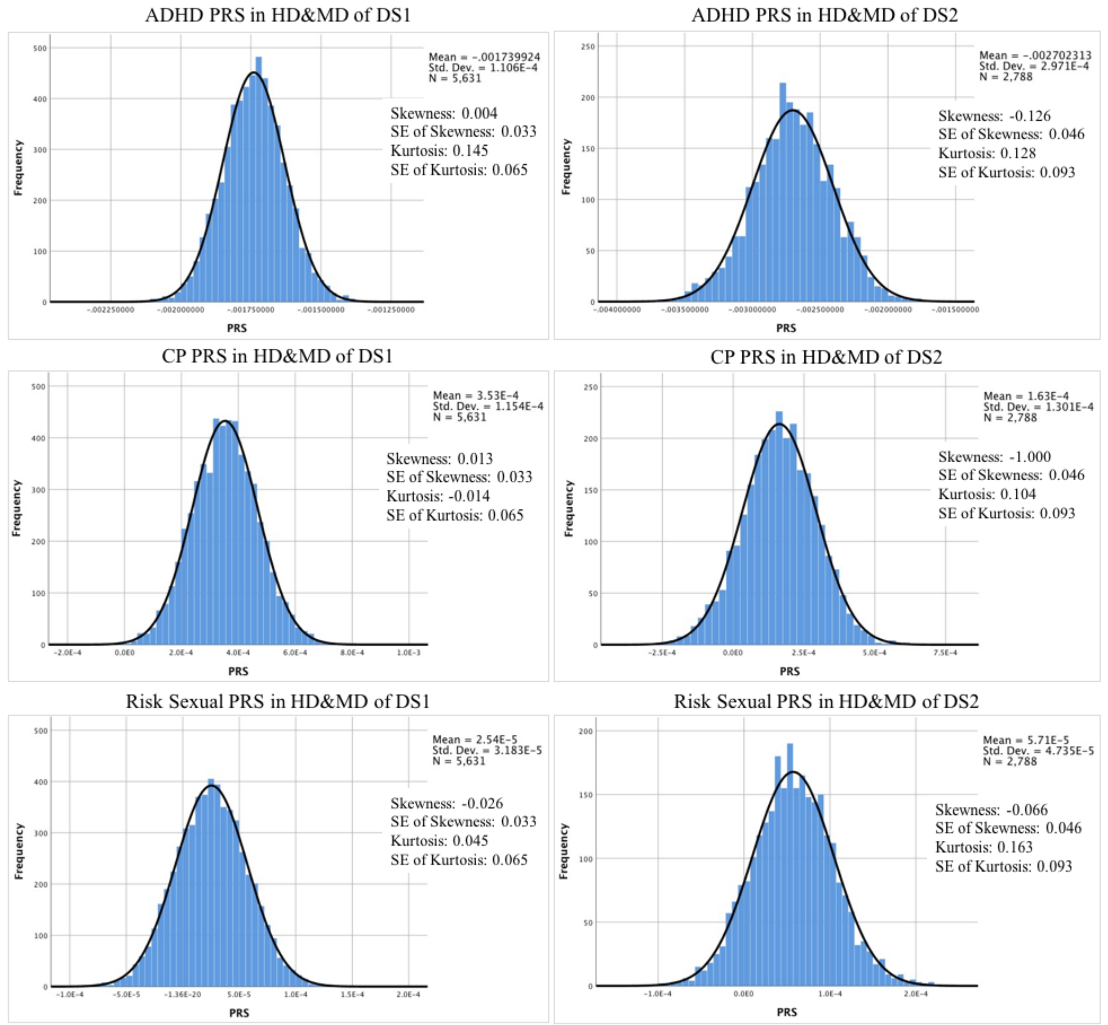

Supplementary tables

Table S1. Sample cohorts of the two GWAS datasets. The number of samples after quality control are shown.

		Heroin Dependence (HD)		MA Dependence (MD)		Alcohol Dependence (AD)		HD&MD	
		**Case**	**Control**	**Case**	**Control**	**Case**	**Control**	**Case**	**Control**
**Dataset 1 (DS1)**	**N**	1028	2862	1750	2861	537	2862	2770	2861
	**Gender (male%)**	71.98%	64.50%	71.83%	64.50%	100%	64.50%	71.81%	64.50%
	**Age (year ± SD)**	35.59±6.96	34.12±6.42	30.50±7.38	34.12±6.42	45.02±9.16	34.12±6.42	32.38±7.64	34.12±6.42
**Dataset 2 (DS2)**	**N**	980	1111	701	1111	224	1097	1678	1110
	**Gender (male%)**	100%	49.23%	100%	49.23%	100%	48.95%	100%	49.28%
	**Age (year ± SD)**	40.15±7.91	20.44±5.37	36.53±8.58	20.44±5.37	40.89±8.80	20.36±5.22	38.64±8.39	22.11±7.27
**Total**		2008	3973	2451	3972	761	3959	4448	3971

Table S2. Number of SNPs after quality control and after imputation for association analysis.

	**Number of SNPs after QC for imputation**		**Number of SNPs after imputation**		
	**GSA**	**ASA**	**GSA**	**ASA**	**Overlapped SNPs for meta-analysis**
AD	382,252	502,229	7,490,201	8,389,326	6,860,949
HD	383,537	503,065	7,585,870	8,362,492	6,913,522
MD	383,309	503,152	7,625,774	8,342,051	6,916,255
HD&MD	383,799	503,993	7,581,743	8,383,841	6,925,876

Table S3. PRS results using DS1 GWAS summary data as discovery to predict the addiction traits in DS2 (AD, HD, MD), and the opposite direction.

**Base**	**Target**	**Best P-value Threshold**	**PRS.R2**	**Coefficient**	**SE**	**P**	**Num_SNP**	**Empirical-P**
DS1_AD	DS2_AD	5.00E-05	0.0042	18.51	7.10	9.14E-03	81	0.1237
DS1_AD	DS2_HD	0.00025	0.0008	-10.12	5.45	6.33E-02	287	0.4968
DS1_AD	DS2_MD	5.00E-05	0.0015	-4.52	2.14	3.48E-02	81	0.3422
DS1_HD	DS2_AD	0.1504	0.0241	2516.72	613.30	4.07E-05	77787	**6.00E-04**
DS1_HD	DS2_HD	0.38145	0.1148	3598.61	402.74	4.06E-19	142265	**1.00E-04**
DS1_HD	DS2_MD	0.406	0.1699	3878.60	337.05	1.21E-30	146530	**1.00E-04**
DS1_MD	DS2_AD	0.0103	0.0126	741.77	178.19	3.14E-05	8574	**5.00E-04**
DS1_MD	DS2_HD	1	0.1047	8138.41	835.89	2.11E-22	226228	**1.00E-04**
DS1_MD	DS2_MD	0.29465	0.1563	4555.49	391.10	2.35E-31	115326	**1.00E-04**
DS2_AD	DS1_AD	0.3832	0.0011	74.89	41.63	0.0720	134256	0.2568
DS2_AD	DS1_HD	0.4998	0.0071	141.78	31.24	5.65E-06	164604	**1.00E-04**
DS2_AD	DS1_MD	0.4992	0.0019	55.11	21.18	0.0093	164580	**0.0442**
DS2_HD	DS1_AD	0.0004	0.0021	-12.29	4.84	0.0110	973	0.0548
DS2_HD	DS1_HD	0.1431	0.0730	307.19	28.86	1.86E-26	71814	**1.00E-04**
DS2_HD	DS1_MD	0.3403	0.0592	419.08	38.40	9.78E-28	132983	**1.00E-04**
DS2_MD	DS1_AD	0.0003	0.0021	-10.79	4.33	0.0128	531	0.0718
DS2_MD	DS1_HD	0.4990	0.0709	681.92	62.04	4.23E-28	167260	**1.00E-04**
DS2_MD	DS1_MD	0.4600	0.0641	673.43	63.63	3.57E-26	159221	**1.00E-04**

DS1: data set 1; DS2: data set 2; AD: Alcohol dependence; HD: heroin dependence; MD: methamphetamine dependence.

Table S4. Detailed association analysis results of the five significant loci for each type of substance dependence trait in each dataset. AD: Alcohol dependence, HD: heroin dependence, MD: MA dependence, HD&MD: combined HD, and MD. DS1: dataset 1, DS2: dataset 2. Meta: meta-analysis results for dataset 1 and dataset 2.

Locus order	Associated trait	Chr	Referred Genes	Lead SNP (trait)	Pos	A1	A2	Data set	N	MAF	Beta	SE	P-value
1	AD	chr4	ADH1A, ADH1B, ADH1C, ADH6	rs13106840 (AD)	100225459	G	A	AD_DS1	3405	0.2626	-0.787	0.0951	**1.29E-16**
								AD_DS2	1321	0.2966	-1.6113	0.367	1.13E-05
								AD_Meta			-0.8378	0.0921	**9.68E-20**
								HD_DS1	3887	0.2158	0.0566	0.0935	0.5447
								HD_DS2	2091	0.2002	0.2635	0.1708	0.1228
								HD_Meta			0.1057	0.0859	0.2187
								MD_DS1	4608	0.2129	0.081	0.0696	0.2444
								MD_DS2	1812	0.2064	0.1849	0.1493	0.2156
								MD_Meta			0.0999	0.064	0.1189
								HD&MD_DS1	5629	0.2054	0.0726	0.0644	0.2596
								HD&MD_DS2	2788	0.1911	0.19	0.1415	0.1796
								HD&MD_Meta			0.0929	0.0589	0.1148
2	AD	chr12	BRAP, ALDH2, ACAD10, ADAM1A, ERP29, TRAFD1, MAPKAPK5, TMEM116,	rs671 (AD)	112241766	G	A	AD_DS1	3405	0.168	-0.8848	0.1478	**2.15E-09**
								AD_DS2	1321	0.1499	-1.3627	0.582	0.0192
								AD_Meta			-0.9131	0.1434	**1.89E-10**
								HD_DS1	3887	0.2206	0.3264	0.0848	1.18E-04
								HD_DS2	2091	0.2374	0.3973	0.1587	1.23E-02
								HD_Meta			0.3426	0.0784	1.23E-05

			NAA25, HECTD4, MIR6761, MIR6861					MD_DS1	4608	0.2153	0.1091	0.0686	0.112
								MD_DS2	1812	0.2238	0.5293	0.1377	1.22E-04
								MD_Meta			0.194	0.0624	1.86E-03
								HD&MD_DS1	5629	0.2311	0.1343	0.063	3.29E-02
								HD&MD_DS2	2788	0.2541	0.4355	0.1318	9.55E-04
								HD&MD_Meta			0.1908	0.0571	8.34E-04
3	HD, MD, HD&MD	chr12	ANKS1B	rs112706556 (HD)	99888384	A	G	AD_DS1	3405	0.1175	0.3676	0.1572	0.0193336
								AD_DS2	1321	0.1996	-0.0688	0.4614	0.881525
								AD_Meta			0.1489	0.03	0.3232
								HD_DS1	3887	0.1138	0.5351	0.1345	6.92E-05
								HD_DS2	2091	0.1758	0.738	0.1775	3.23E-05
								HD_Meta			0.6107	0.112	**4.99E-08**
								MD_DS1	4608	0.1115	0.3602	0.1005	3.37E-04
								MD_DS2	1812	0.1842	0.7919	0.1576	5.00E-07
								MD_Meta			0.4867	0.0859	**1.46E-08**
								HD&MD_DS1	5629	0.1094	0.364	0.0942	1.12E-04
								HD&MD_DS2	2788	0.171	0.7033	0.1498	2.67E-06
								HD&MD_Meta			0.4611	0.0801	**8.70E-09**
4	HD&MD	chr2	NRXN1	rs74330628 (HD&MD)	50041088	A	G	AD_DS1	3405	0.1093	0.213	0.1771	0.2292
								AD_DS2	1321	0.1166	-0.5886	0.7212	0.4144
								AD_Meta			0.1684	0.1721	0.328
								HD_DS1	3887	0.1098	-0.3478	0.1363	1.08E-02
								HD_DS2	2091	0.124	-1.0015	0.2479	5.36E-05
								HD_Meta			-0.5038	0.1251	5.68E-05

								MD_DS1	4608	0.1082	-0.3897	0.1061	2.40E-04
								MD_DS2	1812	0.124	-0.8344	0.2075	5.81E-05
								MD_Meta			-0.4834	0.0959	4.64E-07
								HD&MD_DS1	5629	0.1088	-0.3976	0.0984	5.37E-05
								HD&MD_DS2	2788	0.1242	-0.8687	0.2019	1.69E-05
								HD&MD_Meta			-0.489	0.0889	**3.84E-08**
5	HD&MD	chr7	GTF2IRD1	rs76965632 (HD&MD)	73938239	C	T	AD_DS1	3405	0.0183	-0.5601	0.3769	0.1373
								AD_DS2	1321	0.0375	-0.269	0.9984	0.7876
								AD_Meta			-0.5245	0.3531	0.1374
								HD_DS1	3887	0.0209	-0.8847	0.2943	2.65E-03
								HD_DS2	2091	0.0347	-1.6166	0.3576	6.18E-06
								HD_Meta			-1.1865	0.2373	5.73E-07
								MD_DS1	4608	0.0185	-0.8293	0.2525	1.02E-03
								MD_DS2	1812	0.0337	-1.4478	0.3299	1.14E-05
								MD_Meta			-1.0607	0.2032	1.78E-07
								HD&MD_DS1	5629	0.0191	-0.8007	0.2254	3.83E-04
								HD&MD_DS2	2788	0.0325	-1.5931	0.3274	1.14E-06
								HD&MD_Meta			-1.0578	0.1865	**1.41E-08**

AD: Alcohol dependence, HD: heroin dependence, MD: MA dependence, HD&MD: combined HD and MD. DS1: data set1, DS2: data set 2, Meta: meta-analysis result for data set 1 and data set 2.

Table S5. eQTL data for the SNPs in LD with the lead SNP of *ANKS1B* locus and *NRXN1* locus in 13 brain regions in the GTEx v8 database. Bonferroni corrected *P*-value < 0.05/13 = 0.00385 was considered to be significant.

Gene Symbol	SNP	P-Value	NES	T-statistic	Tissue
ANKS1B	rs7968525	0.00055	-0.4	-3.6	Brain - Spinal cord (cervical c-1)
ANKS1B	rs12580184	0.0035	-0.35	-3	Brain - Spinal cord (cervical c-1)
ANKS1B	rs17029487	0.0035	-0.35	-3	Brain - Spinal cord (cervical c-1)
ANKS1B	rs58720542	0.00055	-0.4	-3.6	Brain - Spinal cord (cervical c-1)
ANKS1B	rs2133896	0.0035	-0.35	-3	Brain - Spinal cord (cervical c-1)
NRXN1	rs1363047	0.000044	0.2	4.2	Brain - Anterior cingulate cortex (BA24)
NRXN1	rs150598003	0.000058	0.19	4.2	Brain - Anterior cingulate cortex (BA24)
NRXN1	rs1836546	0.000062	0.19	4.1	Brain - Anterior cingulate cortex (BA24)
NRXN1	rs77953432	0.000084	0.19	4.1	Brain - Anterior cingulate cortex (BA24)
NRXN1	rs77729728	0.000088	0.19	4.1	Brain - Anterior cingulate cortex (BA24)
NRXN1	rs113222369	0.00014	0.19	3.9	Brain - Anterior cingulate cortex (BA24)
NRXN1	rs77913980	0.00014	0.19	3.9	Brain - Anterior cingulate cortex (BA24)
NRXN1	rs377055079	0.00019	0.18	3.9	Brain - Anterior cingulate cortex (BA24)
NRXN1	rs72831199	0.0015	0.15	3.2	Brain - Anterior cingulate cortex (BA24)
NRXN1	rs55903729	0.0026	0.077	3.1	Brain - Hippocampus
NRXN1	rs11693780	0.0026	0.077	3.1	Brain - Hippocampus

Table S6. Association of the three significant loci of HD&MD with three addiction-related phenotypes. Considering the risk SNP will lead to more severe craving, dose, and frequency, a one-tail p-value was used. The *P*-value from the meta-analysis was adjusted using P_{meta}*9.

CHR	Gene annotation	SNP	BP	A1	Phenotype	DS1			DS2			Meta			
						**BETA**	**SE**	**P**	**BETA**	**SE**	**P**	**BETA**	**SE**	**P**	**adjusted P**
2	NRXN1	rs74330628	50041088	A	MA Craving	-0.0702	0.1828	0.3506	-0.2062	0.2551	0.2096	-0.1163	0.1486	0.2169	1
					MA Dose	0.0629	0.0410	0.0623	0.0322	0.0715	0.3261	0.0553	0.0355	0.0597	0.5373
					Heroin Frequency	-0.5059	0.2439	**0.0192**	-0.3396	0.1611	**0.0177**	-0.3901	0.1344	**0.0019**	**0.0167**
7	GTF2IRD1	rs76965632	73938239	C	MA Craving	-0.9721	0.4099	**0.0089**	-0.6303	0.4092	0.0620	-0.8009	0.2896	**0.0028**	**0.0256**
					MA Dose	-0.0805	0.0900	0.1859	-0.2742	0.1081	**0.0057**	-0.1598	0.0692	**0.0105**	0.0940
					Heroin Frequency	0.1413	0.5013	0.3890	0.0519	0.2211	0.4073	0.0664	0.2023	0.3713	1
12	ANKS1B	rs10860455	99916089	T	MA Craving	0.1286	0.0966	0.0917	0.0301	0.1681	0.4289	0.1042	0.0837	0.1068	0.9612
					MA Dose	-0.0036	0.0215	0.4328	-0.0908	0.0442	**0.0202**	-0.0203	0.0193	0.1469	1
					Heroin Frequency	0.2906	0.1309	**0.0134**	-0.0601	0.1024	0.2787	0.0730	0.0807	0.1827	1

DS1: data set1, DS2: data set 2, Meta: meta-analysis result for data set 1 and data set 2. MA: methamphetamine. P<0.05 was highlighted in bold.

Table S7. Association analysis for the SNPs of the significant loci of HD&MD with EverDrugs of the HCP dataset.

SNP	Beta	SE	P-value
chr12_rs10860447	0.2242	0.1242	**0.0355**
chr12_rs2202037	0.2236	0.1252	**0.0370**
chr12_rs1912861	0.2071	0.1256	**0.0496**
chr12_rs78362061	0.1581	0.1812	0.1914
chr2_rs6750490	-0.0636	0.1422	0.3273
chr2_rs1363047	-0.0522	0.1305	0.3444
chr7_rs76965632	-12.3235	535.4112	0.4908

Table S8. Association analysis for the brain regions with EverDrugs of the HCP dataset.

**Brain name**	**Beta**	**SE**	**p-value**
**FS_L_Amygdala_Vol**	**-31.0480**	**10.7545**	**0.0040**
**FS_R_Amygdala_Vol**	**-26.6122**	**11.8631**	**0.0251**
FS_L_ThalamusProper_Vol	-40.5877	48.9339	0.4070
FS_R_ThalamusProper_Vol	-90.6545	42.2802	0.0322
FS_L_Pallidum_Vol	1.3086	15.5422	0.9329
FS_R_Pallidum_Vol	15.4506	12.1606	0.2042
FS_L_Hippo_Vol	-33.4877	27.9439	0.2310
FS_R_Hippo_Vol	-22.4696	25.0168	0.3693
FS_L_AccumbensArea_Vol	1.9162	5.8592	0.7437
FS_R_AccumbensArea_Vol	-1.9986	6.2615	0.7496
FS_L_Caudate_Vol	2.0715	28.4733	0.9420
FS_R_Caudate_Vol	-5.6541	28.6145	0.8434
FS_L_Putamen_Vol	-21.5929	46.0761	0.6394
FS_R_Putamen_Vol	-0.4607	37.3399	0.9902

Table S9. *ANKS1B* associated traits from PheWAS analysis using the GWAS Atlas. Number of GWASs considered: 4756 (including GWASs in which the searched SNP or gene was not tested, Bonferroni corrected P-value: 1.05e-5).

Atlas ID	PMID	Year	Domain	Trait	P-value	N
4074	30239722	2018	Metabolic	Body Mass Index	2.11E-25	806834
3435	31427789	2019	Metabolic	Body Mass Index	3.87E-18	385336
3445	31427789	2019	Metabolic	Impedance measures - Body Mass Index (BMI)	7.30E-18	379831
3436	31427789	2019	Metabolic	Weight	3.58E-15	385473
3457	31427789	2019	Metabolic	Impedance measures - Leg fat mass (left)	4.04E-15	379783
3453	31427789	2019	Metabolic	Impedance measures - Leg fat mass (right)	8.11E-15	379802
3440	31427789	2019	Metabolic	Impedance measures - Weight	1.36E-14	379840
4044	30124842	2018	Metabolic	Body Mass Index	2.06E-14	681275
4076	30239722	2018	Metabolic	Body Mass Index (female)	3.57E-14	434794
3465	31427789	2019	Metabolic	Impedance measures - Arm fat mass (left)	5.60E-14	379663
3461	31427789	2019	Metabolic	Impedance measures - Arm fat mass (right)	8.96E-14	379725
3442	31427789	2019	Metabolic	Impedance measures - Whole body fat mass	1.00E-13	379203
3460	31427789	2019	Metabolic	Impedance measures - Arm fat percentage (right)	1.73E-13	379752
3464	31427789	2019	Metabolic	Impedance measures - Arm fat percentage (left)	2.69E-13	379699
3456	31427789	2019	Metabolic	Impedance measures - Leg fat percentage (left)	3.77E-13	379786
3186	31427789	2019	Metabolic	Hip circumference	6.93E-13	385887
3452	31427789	2019	Metabolic	Impedance measures - Leg fat percentage (right)	6.98E-13	379806
3447	31427789	2019	Metabolic	Impedance measures - Impedance of whole body	1.31E-12	379792
4040	29906448	2018	Psychiatric	Schizophrenia/Bipolar disorder	5.97E-12	107620
3446	31427789	2019	Metabolic	Impedance measures - Basal metabolic rate	1.31E-11	379821
3469	31427789	2019	Metabolic	Impedance measures - Trunk fat mass	1.91E-11	379578
3441	31427789	2019	Metabolic	Impedance measures - Body fat percentage	2.11E-11	379615
3449	31427789	2019	Metabolic	Impedance measures - Impedance of leg (left)	3.41E-11	379807
3462	31427789	2019	Metabolic	Impedance measures - Arm fat-free mass (right)	5.42E-11	379723
3467	31427789	2019	Metabolic	Impedance measures - Arm predicted mass (left)	5.68E-11	379638
3459	31427789	2019	Metabolic	Impedance measures - Leg predicted mass (left)	5.73E-11	379761
3443	31427789	2019	Metabolic	Impedance measures - Whole body fat-free mass	5.97E-11	379804
3444	31427789	2019	Metabolic	Impedance measures - Whole body water mass	6.99E-11	379835
3458	31427789	2019	Metabolic	Impedance measures - Leg fat-free mass (left)	1.01E-10	379766
3982	29483656	2018	Psychiatric	Schizophrenia	1.24E-10	105318
3270	31427789	2019	Metabolic	Comparative body size at age 10	1.34E-10	379749
3466	31427789	2019	Metabolic	Impedance measures - Arm fat-free mass (left)	1.59E-10	379653
3463	31427789	2019	Metabolic	Impedance measures - Arm predicted mass (right)	1.65E-10	379716
4302	30664634	2019	Metabolic	Arms-arm fat ratio (female)	2.59E-10	195068
3471	31427789	2019	Metabolic	Impedance measures - Trunk predicted mass	4.75E-10	379469
4075	30239722	2018	Metabolic	Body Mass Index (male)	7.07E-10	374756
3470	31427789	2019	Metabolic	Impedance measures - Trunk fat-free mass	8.08E-10	379507
3185	31427789	2019	Metabolic	Waist circumference	1.37E-09	385932

3454	31427789	2019	Metabolic	Impedance measures - Leg fat-free mass (right)	2.32E-09	379793
3291	31427789	2019	Psychiatric	Tense / 'highly strung'	2.45E-09	374129
4166	30108127	2018	Metabolic	Body Mass Index	2.70E-09	334487
3455	31427789	2019	Metabolic	Impedance measures - Leg predicted mass (right)	2.87E-09	379793
3448	31427789	2019	Metabolic	Impedance measures - Impedance of leg (right)	3.25E-09	379813
3998	29500382	2018	Psychiatric	Tense / 'highly strung' (TENSE)	4.35E-09	263635
3798	29942085	2018	Psychiatric	Worry subcluster	7.11E-09	348219
11	25056061	2014	Psychiatric	Schizophrenia	9.74E-09	82315
13	24280982	2014	Psychiatric	Schizophrenia vs Bipolar disorder	9.74E-09	16381
3468	31427789	2019	Metabolic	Impedance measures - Trunk fat percentage	1.03E-08	379600
3451	31427789	2019	Metabolic	Impedance measures - Impedance of arm (left)	1.13E-08	379803
3996	29500382	2018	Psychiatric	Nervous feelings (NERV-FEEL)	1.41E-08	264858
3450	31427789	2019	Metabolic	Impedance measures - Impedance of arm (right)	1.46E-08	379786
3511	31427789	2019	Psychiatric	Reason for reducing amount of alcohol drunk: Other reason	3.17E-08	142645
3295	31427789	2019	Psychiatric	Guilty feelings	4.47E-08	376361
3289	31427789	2019	Psychiatric	Nervous feelings	9.89E-08	376368
4002	29500382	2018	Psychiatric	Guilty feelings (GUILT)	2.01E-07	265139
4293	30718901	2019	Psychiatric	Depression	2.56E-07	500199
3785	29942086	2018	Cognitive	Intelligence	7.28E-07	269867
1191	23453885	2013	Psychiatric	PGC cross disorder	7.37E-07	61220
4038	29906448	2018	Psychiatric	Schizophrenia	9.22E-07	87491
3301	31427789	2019	Psychiatric	Seen doctor (GP) for nerves, anxiety, tension or depression	9.72E-07	383771
3307	31427789	2019	Activities	Overall health rating	1.02E-06	384850
3990	29500382	2018	Psychiatric	Neuroticism sum score	1.47E-06	380506
4071	BioRxiv: https://doi.org/10.1101/261081	2018	Reproduction	Number of sexual partners	1.75E-06	370711
2017	24369049	2014	Psychiatric	Lithium response in Bipolar I patients - Alda Scale of 6 to 7	0.000002984	294
3212	31427789	2019	Activities	Frequency of walking for pleasure in last 4 weeks	4.28E-06	275324
3250	31427789	2019	Nutritional	Pork intake	5.40E-06	384328
12	24280982	2014	Psychiatric	Schizophrenia/Bipolar disorder	5.89E-06	39202
3290	31427789	2019	Psychiatric	Worrier / anxious feelings	0.000007104	376411
1731	17903292	2007	Metabolic	Chronic kidney disease (at exam 7, GEE, adjusted for age and sex)	8.98E-06	1010

Table S10. NRXN1 associated traits from PheWAS analysis using the GWAS Atlas. Number of GWASs considered: 4756 (including GWASs in which the searched SNP or gene was not tested, Bonferroni corrected P-value: 1.05e-5).

Atlas ID	PMID	Year	Domain	Trait	P-value	N
4074	30239722	2018	Metabolic	Body Mass Index	1.69E-34	806834
3456	31427789	2019	Metabolic	Impedance measures - Leg fat percentage (left)	1.76E-25	379786
4044	30124842	2018	Metabolic	Body Mass Index	6.58E-25	681275
3445	31427789	2019	Metabolic	Impedance measures - Body Mass Index (BMI)	9.16E-25	379831
3457	31427789	2019	Metabolic	Impedance measures - Leg fat mass (left)	6.16E-24	379783
3435	31427789	2019	Metabolic	Body Mass Index	1.83E-23	385336
3452	31427789	2019	Metabolic	Impedance measures - Leg fat percentage (right)	3.46E-23	379806
3453	31427789	2019	Metabolic	Impedance measures - Leg fat mass (right)	4.79E-23	379802
3442	31427789	2019	Metabolic	Impedance measures - Whole body fat mass	6.55E-23	379203
3440	31427789	2019	Metabolic	Impedance measures - Weight	1.70E-22	379840
3441	31427789	2019	Metabolic	Impedance measures - Body fat percentage	3.04E-22	379615
3469	31427789	2019	Metabolic	Impedance measures - Trunk fat mass	8.78E-22	379578
3461	31427789	2019	Metabolic	Impedance measures - Arm fat mass (right)	1.34E-21	379725
3436	31427789	2019	Metabolic	Weight	1.59E-21	385473
4066	30038396	2018	Environment	Educational attainment	1.66E-21	766345
3465	31427789	2019	Metabolic	Impedance measures - Arm fat mass (left)	7.58E-21	379663
3464	31427789	2019	Metabolic	Impedance measures - Arm fat percentage (left)	1.01E-20	379699
3460	31427789	2019	Metabolic	Impedance measures - Arm fat percentage (right)	2.35E-20	379752
3468	31427789	2019	Metabolic	Impedance measures - Trunk fat percentage	2.30E-19	379600
4076	30239722	2018	Metabolic	Body Mass Index (female)	2.36E-19	434794
3467	31427789	2019	Metabolic	Impedance measures - Arm predicted mass (left)	2.40E-18	379638
3463	31427789	2019	Metabolic	Impedance measures - Arm predicted mass (right)	5.38E-18	379716
3462	31427789	2019	Metabolic	Impedance measures - Arm fat-free mass (right)	1.67E-17	379723
3466	31427789	2019	Metabolic	Impedance measures - Arm fat-free mass (left)	3.80E-17	379653
3446	31427789	2019	Metabolic	Impedance measures - Basal metabolic rate	3.59E-16	379821
3185	31427789	2019	Metabolic	Waist circumference	4.36E-16	385932
3791	30804565	2019	Psychiatric	Ease of getting up in the morning	4.45E-16	385949
3186	31427789	2019	Metabolic	Hip circumference	4.76E-16	385887
3219	31427789	2019	Activities	Time spent watching television (TV)	6.69E-16	365236
3229	31427789	2019	Psychiatric	Getting up in morning	9.88E-16	385494
3443	31427789	2019	Metabolic	Impedance measures - Whole body fat-free mass	8.90E-14	379804
4075	30239722	2018	Metabolic	Body Mass Index (male)	9.86E-14	374756
3444	31427789	2019	Metabolic	Impedance measures - Whole body water mass	1.66E-13	379835

4294	30696823	2019	Psychiatric	Chronotype	1.66E-13	449732
3458	31427789	2019	Metabolic	Impedance measures - Leg fat-free mass (left)	5.53E-13	379766
3459	31427789	2019	Metabolic	Impedance measures - Leg predicted mass (left)	8.41E-13	379761
3789	30804565	2019	Psychiatric	Morningness	3.14E-12	345552
3307	31427789	2019	Activities	Overall health rating	3.52E-12	384850
3230	31427789	2019	Psychiatric	Morning/evening person (chronotype)	4.17E-12	345148
3455	31427789	2019	Metabolic	Impedance measures - Leg predicted mass (right)	6.00E-12	379793
3454	31427789	2019	Metabolic	Impedance measures - Leg fat-free mass (right)	7.58E-12	379793
3450	31427789	2019	Metabolic	Impedance measures - Impedance of arm (right)	1.51E-11	379786
3451	31427789	2019	Metabolic	Impedance measures - Impedance of arm (left)	2.60E-11	379803
3797	29942085	2018	Psychiatric	Depressive affect subcluster	3.40E-11	357957
3409	31427789	2019	Environment	Education - Qualifications	3.68E-11	318526
3379	31427789	2019	Cardiovascular	Diastolic Blood Pressure (automated reading)	4.97E-11	361411
3470	31427789	2019	Metabolic	Impedance measures - Trunk fat-free mass	5.23E-11	379507
4295	30696823	2019	Psychiatric	Morning person (binary)	5.65E-11	403195
3471	31427789	2019	Metabolic	Impedance measures - Trunk predicted mass	6.67E-11	379469
3300	31427789	2019	Psychiatric	Frequency of tiredness / lethargy in last 2 weeks	1.43E-10	375053
3298	31427789	2019	Psychiatric	Frequency of unenthusiasm / disinterest in last 2 weeks	2.92E-10	373833
3254	31427789	2019	Nutritional	Salt added to food	3.08E-10	386322
4378	30940143	2019	Cardiovascular	High blood pressure	3.72E-10	458554
3939	28892062	2017	Metabolic	Body Mass Index	9.14E-10	158284
3415	31427789	2019	Cognitive	Reaction time test - Mean time to correctly identify matches	9.23E-10	383748
4070	BioRxiv: https://doi.org/ 10.1101/261081	2018	Psychiatric	Ever smoker	9.63E-10	518633
4302	30664634	2019	Metabolic	Arms-arm fat ratio (female)	1.09E-09	195068
3285	31427789	2019	Psychiatric	Miserableness	1.14E-09	379907
3380	31427789	2019	Cardiovascular	Systolic Blood Pressure (automated reading)	1.87E-09	361402
3304	31427789	2019	Reproduction	Age first had sexual intercourse	1.88E-09	339614
3992	29500382	2018	Psychiatric	Miserableness (MIS)	2.07E-09	267050
3312	31427789	2019	Activities	Electronic device use - Plays computer games	4.17E-09	386152
3288	31427789	2019	Psychiatric	Fed-up feelings	4.39E-09	378357
3995	29500382	2018	Psychiatric	Fed-up feelings (FED_UP)	4.48E-09	266208
4077	30239722	2018	Metabolic	Waist-hip ratio	4.81E-09	697734
3447	31427789	2019	Metabolic	Impedance measures - Impedance of whole body	9.29E-09	379792
3578	31427789	2019	Social Interactions	Social support - Leisure/social activities: Religious group	1.18E-08	385280
4327	30643256	2019	Psychiatric	Well-being spectrum	1.27E-08	2311184
3796	29942085	2018	Psychiatric	Depressive symptoms	1.36E-08	381455

4087	29255261	2018	Psychiatric	Neuroticism	3.70E-08	329821
3990	29500382	2018	Psychiatric	Neuroticism sum score	8.79E-08	380506
3267	31427789	2019	Psychiatric	Alcohol usually taken with meals	1.16E-07	197613
42	27864402	2016	Activities	Self-rated health	1.25E-07	111749
3308	31427789	2019	Mortality	Long-standing illness, disability or infirmity	1.29E-07	377498
4174	29970889	2018	Social Interactions	Social support - Leisure/social activities: Religious group	1.50E-07	452302
3941	28892062	2017	Metabolic	Body Mass Index (female)	2.45E-07	72390
3982	29483656	2018	Psychiatric	Schizophrenia	3.89E-07	105318
4067	30038396	2018	Cognitive	Cognitive performance	3.89E-07	257828
4307	30664745	2019	Skeletal	Osteoarthritis	4.56E-07	455221
4269	30867560	2019	Psychiatric	Neuroticism general factor	5.86E-07	270059
3210	31427789	2019	Activities	Usual walking pace	6.08E-07	384081
3795	29942085	2018	Psychiatric	Neuroticism	8.44E-07	390278
3295	31427789	2019	Psychiatric	Guilty feelings	9.40E-07	376361
3297	31427789	2019	Psychiatric	Frequency of depressed mood in last 2 weeks	9.57E-07	370017
3294	31427789	2019	Psychiatric	Loneliness, isolation	1.15E-06	380317
3551	31427789	2019	Cardiovascular	Vascular/heart problems diagnosed by doctor: High blood pressure	1.21E-06	385699
3478	31427789	2019	Activities	Own or rent accommodation lived in: Rent - from local authority, local council, housing association	1.23E-06	383032
2018	24369049	2014	Psychiatric	Lithium response in Bipolar I patients - Alda Scale of 7 to 8	1.35E-06	294
2024	23089632	2013	Psychiatric	Alcohol dependence	1.58E-06	2322
3218	31427789	2019	Activities	Time spent outdoors in winter	1.62E-06	304316
4002	29500382	2018	Psychiatric	Guilty feelings (GUILT)	1.89E-06	265139
4001	29500382	2018	Psychiatric	Loneliness, isolation (LONE)	2.35E-06	267190
4314	30643251	2019	Psychiatric	Ever smoked regulary	2.89E-06	262990
4092	29844566	2018	Cognitive	Reaction time	2.90E-06	330069
3203	31427789	2019	Environment	Age completed full time education	2.94E-06	253580
3785	29942086	2018	Cognitive	Intelligence	2.95E-06	269867
2015	24369049	2014	Psychiatric	Lithium response in Bipolar I patients - Alda Scale of 4 to 5	2.96E-06	294
3235	31427789	2019	Psychiatric	Current tobacco smoking	3.32E-06	386150
45	28194004	2018	Psychiatric	Self-reported tiredness	3.47E-06	108976
2007	22377632	2012	Reproduction	Parental transmission distortion	3.67E-06	4728
2016	24369049	2014	Psychiatric	Lithium response in Bipolar I patients - Alda Scale of 5 to 6	3.96E-06	294
3448	31427789	2019	Metabolic	Impedance measures - Impedance of leg (right)	4.66E-06	379813
4272	30846698	2019	Psychiatric	Sleep duration	4.77E-06	446118
53	27225129	2016	Environment	Educational attainment	4.95E-06	328917
3449	31427789	2019	Metabolic	Impedance measures - Impedance of leg (left)	5.67E-06	379807
3499	31427789	2019	Dermatological	Hair colour (natural, before greying): Black	5.84E-06	385603
3188	31427789	2019	Cardiovascular	Pulse rate (automated reading)	6.00E-06	361411
3494	31427789	2019	Nutritional	Coffee type: Ground coffee (include espresso, filter etc)	6.07E-06	303811

4228	31015401	2019	Environmental	Anti-inflammatory and antirheumatic products, non-steroids	6.60E-06	164520
3253	31427789	2019	Nutritional	Cereal intake	8.13E-06	368775
3417	31427789	2019	Psychiatric	Neuroticism score	8.44E-06	312740
3558	31427789	2019	Activities	Medication for pain relief, constipation, heartburn: Ibuprofen (e.g. Nurofen)	8.49E-06	382089
3315	31427789	2019	Activities	Use of sun/uv protection	8.64E-06	383659
2005	22377632	2012	Reproduction	Maternal transmission distortion	8.76E-06	4728
2006	22377632	2012	Reproduction	Paternal transmission distortion	8.76E-06	4728
3654	31427789	2019	Psychiatric	Smoking status: Never	9.80E-06	384964

Table S11. GTF2IRD1 associated traits from PheWAS analysis using the GWAS Atlas. Number of GWASs considered: 4756 (including GWASs in which the searched SNP or gene was not tested, Bonferroni corrected P-value: 1.05e-5).

Atlas ID	PMID	Year	Domain	Trait	P-value	N
4269	30867560	2019	Psychiatric	Neuroticism general factor	3.14E-10	270059
4087	29255261	2018	Psychiatric	Neuroticism	5.99E-09	329821
4327	30643256	2019	Psychiatric	Well-being spectrum	2.90E-07	2311184
4328	30598549	2018	Skeletal	Estimated bone mineral density from heel ultrasounds	1.2708E-06	426824
4293	30718901	2019	Psychiatric	Depression	1.3918E-06	500199
4043	30124842	2018	Skeletal	Height	2.0531E-06	693529
4321	30643256	2019	Psychiatric	Neuroticism (univariate)	3.7054E-06	523783
3417	31427789	2019	Psychiatric	Neuroticism score	7.1885E-06	312740

Table S12. Gene-level association analysis for AD, HD, MD, and HD&MD using MAGMA.

Trait	GENE	CHR	START	STOP	NSNPS	NPARAM	N	ZSTAT	P	Padj
HD	KIAA1217	10	23983675	24836772	1592	106	5981	5.6651	7.35E-09	1.27E-04
	FAM19A5	22	48885288	49147744	830	109	5981	5.6568	7.71E-09	1.34E-04
	NAV3	12	78224685	78606790	682	52	5981	5.5863	1.16E-08	2.01E-04
	TMEM132D	12	129556271	130388212	1870	211	5981	5.2342	8.29E-08	1.44E-03
	**GRM7**	3	6902802	7783218	1616	138	5981	5.2126	9.31E-08	1.62E-03
	RGS7	1	240938817	241520505	845	93	5981	5.0988	1.71E-07	2.96E-03
	**RBFOX1**	16	5289469	7763342	6748	490	5981	4.9862	3.08E-07	5.34E-03
	NFIA	1	61542946	61928460	515	67	5981	4.9548	3.62E-07	6.28E-03
	**CDH13**	16	82660399	83830215	3275	263	5981	4.9178	4.38E-07	7.59E-03
	LAPTM5	1	31205315	31230683	31	9	5981	4.8079	7.63E-07	0.0132
	MARCH1	4	164445450	165305093	1762	88	5981	4.737	1.08E-06	0.0188
	MYRIP	3	39850405	40301812	941	60	5981	4.7367	1.09E-06	0.0188
	KCNN3	1	154669938	154842754	243	49	5981	4.7362	1.09E-06	0.0189
	CNTN4	3	2140550	3099645	1759	188	5981	4.7173	1.20E-06	0.0207
	SPATA13	13	24553765	24881212	820	107	5981	4.6987	1.31E-06	0.0227
	**ANKS1B**	12	99128569	100378512	1265	106	5981	4.6477	1.68E-06	0.0291
	CCDC33	15	74528630	74628813	242	35	5981	4.5562	2.60E-06	0.0452
	CALN1	7	71244476	71912136	986	59	5981	4.5526	2.65E-06	0.0459
MD	**RBFOX1**	16	5289469	7763342	6519	460	6420	5.9558	1.29E-09	2.23E-05
	**ANKS1B**	12	99128569	100378512	1413	115	6420	5.8	3.32E-09	5.72E-05
	**CDH13**	16	82660399	83830215	2965	252	6420	5.0255	2.51E-07	4.33E-03
	**GRM7**	3	6902802	7783218	1728	136	6420	4.9738	3.28E-07	5.67E-03

	SORBS1	10	97071530	97321171	467	48	6420	4.5365	2.86E-06	0.0494
AD	ADH1B	4	100227527	100242572	18	3	4726	7.5759	1.78E-14	3.04E-10
	ADH1A	4	100197523	100212185	25	5	4726	6.8138	4.75E-12	8.10E-08
	ADH4	4	100044832	100065449	14	4	4726	6.1018	5.25E-10	8.94E-06
	ADH5	4	99992129	100009931	9	2	4726	6.0364	7.88E-10	1.34E-05
	ADH6	4	100123795	100140403	18	3	4726	5.6827	6.63E-09	1.13E-04
	ADH1C	4	100257649	100273917	91	4	4726	5.04	2.33E-07	3.97E-03
	NFYB	12	104510858	104532040	10	4	4726	4.761	9.63E-07	0.0164
HD&MD	**CDH13**	16	82660399	83830215	3004	260	8419	5.5834	1.18E-08	2.03E-04
	**ANKS1B**	12	99128569	100378512	1224	111	8419	5.4842	2.08E-08	3.58E-04
	**RBFOX1**	16	5289469	7763342	6755	460	8419	5.4007	3.32E-08	5.73E-04
	FAM19A5	22	48885288	49147744	725	99	8419	5.2386	8.09E-08	1.40E-03
	CSMD1	8	2792875	4852328	8865	474	8419	4.7654	9.42E-07	0.0163
	**GRM7**	3	6902802	7783218	1708	125	8419	4.6125	1.99E-06	0.0343
	SORCS2	4	7194374	7744564	1402	178	8419	4.6123	1.99E-06	0.0344
	MYH13	17	10201401	10276322	200	27	8419	4.5727	2.41E-06	0.0415
	SORBS1	10	97071530	97321171	462	47	8419	4.5526	2.65E-06	0.0457

AD: Alcohol dependence, HD: heroin dependence, MD: MA dependence, HD&MD: combined HD and MD.

Table S13. GWAS summary data for polygenic risk score analysis as the discovery data set.

Group	Data set	Reference	Sample size[a]	Number of SNPs
Addiction related traits	Alcohol dependence in European (AD-EUR)	Raymond K. Walters MJA, Amy E. Adkins, Fazil Aliev, SilviuAlin Bacanu, Anthony Batzler, Sarah Bertelsen, Joanna M. Biernacka, Tim B. Bigdeli, Li Shiun Chen, Toni-Kim Clarke, Yi-Ling Chou. Trans-ancestral GWAS of alcohol dependence reveals common genetic underpinnings with psychiatric disorders. Nat Neurosci. 2018 Dec;21(12):1656-1669.	8,485 cases, 20,272 controls	9,142,833
	Ever smoke	Tobacco, Genetics C. Genome-wide meta-analyses identify multiple loci associated with smoking behavior. Nat Genet. 2010;42(5):441-447.	74,053 individuals	2,455,848
	Cigs per day	Tobacco, Genetics C. Genome-wide meta-analyses identify multiple loci associated with smoking behavior. Nat Genet. 2010;42(5):441-447.	74,053 individuals	2,459,120
	Problematic alcohol use (PAU, PAU_MVP1_MVP2_PGC_UKB_Dec11.txt)	Zhou H, Sealock JM, Sanchez-Roige S, Clarke TK, Levey DF, Cheng Z, et al. Genome-wide meta-analysis of problematic alcohol use in 435,563 individuals yields insights into biology and relationships with other traits. Nature neuroscience. 2020;23(7):809-18.	435,563 individuals	14,069,428
	alcohol use disorder (AUD, AUD_MVP1_MVP2_PGC_Dec11.txt)	Kranzler HR, Zhou H, Kember RL, Vickers Smith R, Justice AC, Damrauer S, et al. Genome-wide association study of alcohol consumption and use disorder in 274,424 individuals from multiple populations. Nature communications. 2019;10(1):1499.	274,424 individuals	7,003,541
	opioid use disorder (OUD, OUD_trans_ancestry_meta_Mar12.beta.SE.txt)	Zhou H, Rentsch CT, Cheng Z, Kember RL, Nunez YZ, Sherva RM, et al. Association of OPRM1 Functional Coding Variant with Opioid Use Disorder: A Genome-Wide Association Study. JAMA psychiatry. 2020;77(10):1072-80.	European American individuals, 10 544 OUD cases and 72 163 opioid-exposed controls; African American individuals, 5212 cases and 26 876 controls	9,415,239
Risky behavior	General risk tolerance	Karlsson Linner R, Biroli P, Kong E, et al. Genome-wide association analyses of risk tolerance and risky behaviors in over 1 million individuals identify hundreds of loci and shared genetic influences. Nat Genet. 2019;51(2):245-257.	466,571 individuals	11,514,382
	Risky speeding	Karlsson Linner R, Biroli P, Kong E, et al. Genome-wide association analyses of risk tolerance and risky behaviors in over 1 million individuals identify hundreds of loci and shared genetic influences. Nat Genet. 2019;51(2):245-257.	404,291 individuals	11,514,464

	Risky drink	Karlsson Linner R, Biroli P, Kong E, et al. Genome-wide association analyses of risk tolerance and risky behaviors in over 1 million individuals identify hundreds of loci and shared genetic influences. Nat Genet. 2019;51(2):245-257.	414,343 individuals	11,514,937
	Risky smoke	Karlsson Linner R, Biroli P, Kong E, et al. Genome-wide association analyses of risk tolerance and risky behaviors in over 1 million individuals identify hundreds of loci and shared genetic influences. Nat Genet. 2019;51(2):245-257.	518,633 individuals	11,514,655
	Risky sex	Karlsson Linner R, Biroli P, Kong E, et al. Genome-wide association analyses of risk tolerance and risky behaviors in over 1 million individuals identify hundreds of loci and shared genetic influences. Nat Genet. 2019;51(2):245-257.	370,711 individuals	11,515,110
Cognition	Educational attainment	Lee JJ, Wedow R, Okbay A, et al. Gene discovery and polygenic prediction from a genome-wide association study of educational attainment in 1.1 million individuals. Nat Genet. 2018;50(8):1112-1121.	1,131,881 individuals	10,101,242
	Cognitive performance	Lee JJ, Wedow R, Okbay A, et al. Gene discovery and polygenic prediction from a genome-wide association study of educational attainment in 1.1 million individuals. Nat Genet. 2018;50(8):1112-1121.	257,841 individuals	10,098,325
	Intelligence	Savage JE, Jansen PR, Stringer S, et al. Genome-wide association meta-analysis in 269,867 individuals identifies new genetic and functional links to intelligence. Nat Genet. 2018;50(7):912-919.	269,867 individuals	9,524,855
Psychiatric Disorders	Attention deficit hyperactivity disorder	Demontis D, Walters RK, Martin J, et al. Discovery of the first genome-wide significant risk loci for ADHD. Nat Genet. 2019 Jan;51(1):63-75.	20,183 cases and 35,19[illegible] controls	6,517,325
	Major depressive disorder (MDD.EUR.MVP_UKBB. NatNeuro2021)	Levey DF, Stein MB, Wendt FR, Pathak GA, Zhou H, Aslan M, et al. Bi-ancestral depression GWAS in the Million Veteran Program and meta-analysis in >1.2 million individuals highlight new therapeutic directions. Nature neuroscience. 2021;24(7):954-63.	n = 1,154,267; 340,591 cases	15,636,076
	Bipolar disorder	Sklar P, Ripke S, Scott LJ, et al. Large-scale genome-wide association analysis of bipolar disorder identifies a new susceptibility locus near ODZ4. Nat Genet. 2011;43(10):977-983.	11,974 cases and 51,792 controls	2,427,220
	Schizophrenia	Li Z, Chen J, Yu H, et al. Genome-wide association analysis identifies 30 new susceptibility loci for schizophrenia. Nat Genet. 2017.	42,175 cases and 65,166 controls	4,303,606
	Autism	Anney RJL, Ripke S, Anttila V, et al. Meta-analysis of GWAS of over 16,000 individuals with autism spectrum disorder highlights a novel locus at 10q24.32 and a significant overlap with schizophrenia. Mol Autism. 2017;8.	7,387 cases and 8,567 controls	6,517,325

Anorexia nervosa	Duncan L, Yilmaz Z, Gaspar H, et al. Significant Locus and Metabolic Genetic Correlations Revealed in Genome-Wide Association Study of Anorexia Nervosa. Am J Psychiatry. 2017;174(9):850-858.	3,495 cases and 10,982 controls	10,641,225
Obsessive-compulsive disorder	International Obsessive Compulsive Disorder Foundation Genetics C, Studies OCDCGA. Revealing the complex genetic architecture of obsessive-compulsive disorder using meta-analysis. Mol Psychiatry. 2017.	2,688 cases and 7,037 controls	8,409,518
Tourette Syndrome	Yu D, Sul JH, Tsetsos F, et al. Interrogating the Genetic Determinants of Tourette's Syndrome and Other Tic Disorders Through Genome-Wide Association Studies. Am J Psychiatry. 2019;176(3):217-227.	4,819 cases and 9,488 controls	8,265,320

[a] the sample size was from the original paper. The real sample size for the association analysis of each SNP may not be the same.

Table S14. Polygenic risk score association analysis for PRS of the six addiction-related traits. PAU: Problematic alcohol use, AUD: Alcohol use disorder, OUD: Opioid use disorder, AD: Alcohol dependence, HD: heroin dependence, MD: MA dependence, HD&MD: combined HD and MD. DS1: data set1, DS2: data set 2. Bold numbers denote significant results.

Discovery	Target	PRS result in DS1							Replication in DS2 at the same P-value threshold for significant result in DS1					Meta-analysis result	
		**Threshold**	**No. SNP**	**PRS.R2**	**Estimate**	**SE**	**P**	**Empirical-P**	**R2**	**P**	**Estimate**	**SE**	**No. SNP**	**Estimate**	**SE**
Alcohol dependence		0.4826	103192	0.000643	0.0882	0.052	0.0901	0.6574	0.000015	0.8009	0.0244	0.0969	110136	0.0739	0.0458
Cigs per day		0.00135	840	0.000955	0.1085	0.0525	0.0388	0.3937	0.000577	0.1152	-0.1517	0.0963	824	-0.0088	0.1297
Ever smoke	HD	0.00135	1015	0.002545	0.174	0.0516	0.0008	**0.0191**	0.000574	0.1171	-0.1498	0.0956	1002	0.0221	0.1616
PAU		0.00495	7185	0.000881	0.1028	0.05182	0.0473	0.3941	0.000160	0.4059	-0.03393	0.08488	7167	0.0596	0.0722
AUD		0.2482	56848	0.000362	0.067	0.05263	0.2030	0.8767	0.000893	0.0503	-0.00452	0.08376	54867	0.0780	0.0556
OUD		0.00005	85	0.001904	0.152	0.05218	0.0036	0.0548	0.000314	0.2448	0.06295	0.08458	83	0.0998	0.0360
Alcohol dependence		0.0001	130	0.000571	0.0689	0.0395	0.0811	0.6207	0.000087	0.6055	0.0437	0.0847	146	0.0644	0.0358
Cigs per day		0.0001	83	0.001095	0.095	0.0393	0.0155	0.2011	0.001405	**0.0391**	0.1733	0.084	83	0.1091	0.0356
Ever smoke	MD	0.0002	227	0.000491	0.0644	0.0398	0.1061	0.7367	0.000442	0.2456	-0.1002	0.0863	223	-0.0001	0.0803
PAU		0.00075	2074	0.001585	0.1154	0.0398	0.0037	0.0593	0.000052	0.6894	0.0790	0.0950	2051	0.0975	0.0455
AUD		0.4924	80430	0.001553	0.1155	0.0402	0.0041	0.0625	0.000001	0.9569	-0.1853	0.0947	76112	-0.0469	0.1256
OUD		0.0009	930	0.001388	0.1082	0.0398	0.0066	0.0958	0.000181	0.4567	0.1095	0.0942	938	0.1420	0.0457
Alcohol dependence		5.00E-05	65	0.015798	0.4515	0.0669	1.48E-11	**1.00E-04**	0.002767	**0.0321**	0.6302	0.294	71	0.4598	0.0652
Cigs per day		0.00485	2454	0.00332	0.2032	0.0644	0.0016	**0.0355**	0.000069	0.7256	-0.0704	0.2006	2454	0.1324	0.1199
Ever smoke	AD	5.00E-05	59	0.002035	0.1625	0.0658	0.0136	0.1898	0.001056	0.168	0.4091	0.2968	61	0.1741	0.0643
PAU		5.00E-05	432	0.006244	0.27578	0.0639	1.59E-05	**0.0006**	0.004168	**0.0081**	0.74732	0.2822	450	0.4314	0.2217
AUD		5.00E-05	256	0.006198	0.27467	0.0639	1.71E-05	**0.0009**	0.004041	**0.009**	0.74134	0.2838	268	0.4261	0.2185

OUD		5.00E-05	86	0.001783	0.15191	0.0656	0.0206	0.2345	0.000524	0.3336	0.25116	0.2598	87	0.1579	0.0636
Alcohol dependence		0.0001	131	0.000381	0.0609	0.0366	0.0965	0.6807	0.000218	0.2800	0.0874	0.0809	142	0.0654	0.0333
Cigs per day		0.0001	84	0.000547	0.0728	0.0365	0.0463	0.4550	0.000473	0.1131	0.1313	0.0829	77	0.0823	0.0334
Ever smoke	HD&MD	0.0002	228	0.000847	0.0909	0.0367	0.0132	0.1947	0.000545	0.0877	-0.1379	0.0807	204	-0.0122	0.1138
PAU		0.00075	2069	0.000921	0.0946	0.0366	0.0098	0.1189	0.000105	0.4519	-0.0604	0.0803	1905	0.0336	0.0757
AUD		1	103257	0.000679	0.0818	0.0369	0.0265	0.2595	0.000018	0.7528	-0.0241	0.0766	89823	0.0501	0.0485
OUD		0.0829	32067	0.001034	0.1011	0.0369	0.0062	0.0947	0.000332	0.1815	0.1080	0.0809	29107	0.1022	0.0336

Table S15. Polygenic risk score association analysis for PRS of the five risk traits with substance dependences. AD: Alcohol dependence, HD: heroin dependence, MD: MA dependence, HD&MD: combined HD and MD. DS1: data set1, DS2: data set 2. Bold numbers denote significant results.

**Discovery**	**Target**	**PRS result in DS1**							**Replication in DS2 at the same P-value threshold**				**Meta-analysis result for significant result in DS1**			
		**Threshold**	**No. SNP**	**PRS.R2**	**Estimate**	**SE**	**P**	**Empirical-P**	**R2**	**P**	**Estimate**	**SE**	**No. SNP**	**Estimate**	**SE**	**P**
RiskAll	HD	5.90E-03	8558	0.000328	0.0634	0.0524	0.2260	0.8847	0.001828	0.0053	0.2679	0.0961	8353	0.1498	0.1010	0.1381
RiskDrink		0.0344	26961	0.000572	-0.0829	0.0518	0.1097	0.5916	0.000025	0.7435	-0.0313	0.0958	26294	-0.0712	0.0456	0.1180
RiskSexual		0.0149	18221	0.001499	0.1351	0.0522	0.0096	0.0911	0.002091	0.0028	0.2842	0.0949	17651	0.1885	0.0716	0.0084
RiskSmoke		2.50E-04	3240	0.006882	0.2949	0.0536	3.84E-08	**1.00E-04**	0.000819	0.0606	0.1793	0.0955	3148	0.2641	0.0512	2.48E-07
RiskSpeed		0.0002	1384	0.001642	-0.1416	0.0523	0.0068	0.0795	0.000748	0.0735	-0.1777	0.0993	1381	-0.1497	0.0463	0.0012
RiskAll	MD	0.00765	10030	0.000731	0.0790	0.0401	0.0486	0.3736	0.005817	3.17E-05	0.3644	0.0876	9739	0.2111	0.1423	0.1380
RiskDrink		5.00E-05	815	0.000504	0.0649	0.0396	0.1014	0.5486	0.000068	0.6486	0.0383	0.0840	801	0.0601	0.0358	0.0936
RiskSexual		0.0767	45212	0.006931	0.2452	0.0406	1.48E-09	**1.00E-04**	0.003329	**1.58E-03**	0.2753	0.0871	43335	0.2504	0.0368	**1.02E-11**
RiskSmoke		0.00165	7121	0.008186	0.2693	0.0411	5.38E-11	**1.00E-04**	0.003866	**6.35E-04**	0.2967	0.0868	6895	0.2741	0.0371	**1.61E-13**
RiskSpeed		0.0073	10307	0.000379	-0.0571	0.0402	0.1554	0.7469	0.000071	0.64124	0.0401	0.0860	9974	-0.0382	0.0384	0.3195
RiskAll	AD	1	143175	0.003007	0.1915	0.0639	0.0027	**0.0395**	0.000119	0.6436	0.1076	0.2326	140735	0.1856	0.0616	0.0026
RiskDrink		5.00E-05	804	0.010601	0.3715	0.0664	2.24E-08	**1.00E-04**	0.006270	**1.62E-03**	0.9160	0.2905	798	0.5703	0.2621	**0.0296**
RiskSexual		0.00705	11544	0.000860	-0.1055	0.0655	0.1074	0.5557	0.001277	0.1322	0.3577	0.2376	11571	0.0698	0.2247	0.7560
RiskSmoke		0.02035	23731	0.002606	0.1820	0.0651	0.0052	0.0577	0.000500	0.3425	-0.2245	0.2365	23725	0.0423	0.1930	0.8264
RiskSpeed		0.00925	11618	0.000217	0.0508	0.0629	0.4189	0.9949	0.000105	0.6642	0.1077	0.2481	11544	0.0543	0.0610	0.3734
RiskAll	HD&MD	8.05E-03	10310	0.000327	0.0571	0.0371	0.1236	0.6685	0.001918	0.0014	0.2609	0.0819	9273	0.1459	0.1011	0.1487
RiskDrink		0.00035	1971	0.000266	0.0507	0.0365	0.1645	0.7364	0.000010	0.8177	-0.0186	0.0807	1808	0.0389	0.0333	0.2417
RiskSexual		0.0736	44245	0.004568	0.2138	0.0373	9.87E-09	**1.00E-04**	0.001521	**4.54E-03**	0.2312	0.0815	39580	0.2170	0.0339	**1.57E-10**
RiskSmoke		5.50E-04	4453	0.006663	0.2592	0.0375	4.75E-12	**1.00E-04**	0.002038	**9.87E-04**	0.2623	0.0796	3901	0.2596	0.0339	**1.98E-14**

RiskSpeed	0.00865	11340	0.000357	-0.0595	0.0370	0.1077	0.6194	0.000114	0.4344	0.0626	0.0800	10238	-0.0191	0.0574	0.7394

Table S16. Polygenic risk score association analysis for PRS of the three cognition traits with substance dependences. CP: cognition performance, EA: education attainment, AD: Alcohol dependence, HD: heroin dependence, MD: MA dependence, HD&MD: combined HD and MD. DS1: data set1, DS2: data set 2. Bold numbers denote significant results.

**Discovery**	**Target**	**PRS result in DS1**							**Replication in DS2 at the same P-value threshold for significant result in DS1**				**Meta-analysis result**			
		**Threshold**	**No. SNP**	**PRS.R2**	**Estimate**	**SE**	**P**	**Empirical-P**	**R2**	**P**	**Estimate**	**SE**	**No. SNP**	**Estimate**	**SE**	**P**
CP	HD	0.0017	7360	0.006935	-0.2950	0.0534	3.22E-08	**1.00E-04**	0.001251	**0.021402**	-0.2224	0.0967	7131	-0.2780	0.0467	**2.71E-09**
EA		5.00E-05	3879	0.009359	-0.3475	0.0543	1.54E-10	**1.00E-04**	0.002108	**2.72E-03**	-0.2960	0.0987	3759	-0.3359	0.0476	**1.67E-12**
IQ		0.0033	10482	0.005243	-0.2551	0.0530	1.49E-06	**3.00E-04**	0.001654	**8.05E-03**	-0.2531	0.0955	10137	-0.2546	0.0463	**3.96E-08**
CP	MD	0.00535	12514	0.002526	-0.1456	0.0398	2.50E-04	**4.60E-03**	0.002790	**3.83E-03**	-0.2422	0.0838	12005	-0.1659	0.0389	**2.04E-05**
EA		0.0004	7073	0.010173	-0.3008	0.0411	2.66E-13	**1.00E-04**	0.004184	**3.85E-04**	-0.3115	0.0877	6837	-0.3029	0.0372	**4.02E-16**
IQ		0.00405	11623	0.003906	-0.1814	0.0399	5.44E-06	**4.00E-04**	0.004140	**4.36E-04**	-0.3082	0.0876	11151	-0.2207	0.0589	**1.80E-04**
CP	AD	5.00E-05	1905	0.002477	-0.1771	0.0652	6.55E-03	7.73E-02	0.000049	0.768279	-0.0738	0.2505	1863	-0.1706	0.0631	6.82E-03
EA		0.0388	38334	0.003980	-0.2331	0.0677	5.73E-04	**7.40E-03**	0.002534	0.038127	0.5984	0.2886	38054	0.1353	0.4131	7.43E-01
IQ		0.00475	12222	0.007263	-0.3051	0.0660	3.83E-06	**1.00E-04**	0.000084	0.696713	-0.0937	0.2403	12141	-0.2902	0.0637	5.15E-06
CP	HD&MD	0.0026	8886	0.003260	-0.1782	0.0367	1.23E-06	**1.00E-04**	0.002160	**7.46E-04**	-0.2709	0.0803	7936	-0.1970	0.0374	**1.44E-07**
EA		5.00E-05	3879	0.010274	-0.3263	0.0381	1.07E-17	**1.00E-04**	0.001664	**2.91E-03**	-0.2415	0.0811	3405	-0.3107	0.0345	**2.05E-19**
IQ		0.0035	10812	0.004696	-0.2150	0.0370	6.27E-09	**1.00E-04**	0.001914	**1.46E-03**	-0.2571	0.0808	9586	-0.2223	0.0336	**3.88E-11**

Table S17. Polygenic risk score association analysis for PRS of the eight psychiatric disorders with substance dependencies. ADHD: attention-deficit hyperactivity disorder, ASD: autism disorder, MDD: major depressive disorder, SZ: schizophrenia, BIP: bipolar disorder, TS: Tourette syndrome, OCD: obsessive-compulsive disorder, AN: anorexia nervosa, AD: Alcohol dependence, HD: heroin dependence, MD: MA dependence, HD&MD: combined HD and MD. DS1: data set1, DS2: data set 2. Bold numbers denote significant results.

**Discovery**	**Target**	**PRS result for DS1**							**Replication in DS2 at the same P-value threshold for significant result in DS1**					**Meta-analysis result**		
		**Threshold**	**No. SNP**	**PRS.R2**	**Estimate**	**SE**	**P**	**Empirical-P**	**R2**	**P**	**Estimate**	**SE**	**No. SNP**	**Estimate**	**SE**	**P**
ADHD	HD	0.1509	49483	0.002936	0.1916	0.0530	3.00E-04	**6.00E-03**	0.001319	**0.0178**	0.2370	0.1000	51699	0.2019	0.0468	**1.63E-05**
ASD		0.00045	786	0.000597	0.0844	0.0517	0.1022	0.6451	0.000547	0.1248	0.1443	0.0940	939	0.0983	0.0453	0.0300
MDD		0.3326	97654	0.001439	0.1292	0.0510	0.0112	0.1515	0.000037	0.6907	-0.0385	0.0967	108994	0.0654	0.0813	0.4211
SZ		0.0007	3249	0.000418	0.0733	0.0536	0.1718	0.6394	0.000117	0.4767	0.0701	0.0985	3167	0.0726	0.0471	0.1232
BIP		0.00105	2257	0.000191	0.0480	0.0520	0.3558	0.9812	0.000068	0.5873	0.0515	0.0949	2143	0.0488	0.0456	0.2845
TS		0.0038	3122	0.001435	0.1321	0.0522	0.0114	0.1405	0.000022	0.7603	-0.0285	0.0935	3006	0.0705	0.0780	0.3665
OCD		0.0001	110	0.000663	-0.0887	0.0516	0.0853	0.6242	0.000001	0.9569	0.0053	0.0979	104	-0.0683	0.0456	0.1348
AN		0.0591	23492	0.001163	0.1218	0.0534	0.0226	0.3180	0.000016	0.7920	-0.0260	0.0985	24146	0.0712	0.0703	0.3111
ADHD	MD	0.08135	34139	0.003190	0.1651	0.0401	3.89E-05	**1.40E-03**	0.001798	**0.0195**	0.1992	0.0853	35837	0.1712	0.0363	**2.39E-06**
ASD		0.04765	24698	0.001312	-0.1064	0.0403	0.0082	0.1055	0.000203	0.4318	0.0669	0.0851	28198	-0.0358	0.0849	0.6734
MDD		0.0002	553	0.000542	-0.0673	0.0396	0.0896	0.6442	0.000119	0.5463	-0.0512	0.0849	638	-0.0644	0.0359	0.0726
SZ		0.00535	8362	0.000077	-0.0258	0.0403	0.5215	0.9936	0.000047	0.7049	-0.0328	0.0865	8112	-0.0270	0.0365	0.4592
BIP		0.00455	6071	0.000324	-0.0527	0.0401	0.1887	0.8240	0.000316	0.3259	0.0809	0.0823	5711	-0.0053	0.0639	0.9345
TS		0.0037	3066	0.001713	0.1212	0.0402	0.0026	**4.32E-02**	0.000000	0.9982	0.0002	0.0864	2908	0.0848	0.0553	0.1254
OCD		0.0005	430	0.000260	0.0464	0.0394	0.2392	0.9518	0.000206	0.4273	0.0684	0.0862	405	0.0502	0.0358	0.1612
AN		0.00505	3357	0.001712	0.1204	0.0399	0.0026	0.0537	0.000000	0.9851	-0.0016	0.0852	3513	0.0825	0.0562	0.1419
ADHD	AD	5.00E-05	284	0.000960	-0.1104	0.0649	0.0891	0.5488	0.000071	0.7218	-0.0943	0.2650	325	-0.1095	0.0631	0.0825

ASD		0.007	6030	0.000694	-0.0968	0.0669	0.1479	0.7808	0.001118	0.1649	0.3641	0.2622	7210	0.0639	0.2196	0.7709
MDD		0.00105	1815	0.001400	-0.1306	0.0637	0.0403	0.3783	0.000474	0.3565	0.2343	0.2541	2161	-0.03106	0.1625	0.8484
SZ		0.00085	3465	0.002311	0.1705	0.0647	0.0084	0.0640	0.002785	0.0296	0.6002	0.2760	3451	0.3016	0.1979	0.1275
BIP		0.1945	67052	0.001619	0.1457	0.0661	0.0275	0.2482	0.001040	0.1779	-0.3532	0.2621	63159	-0.0393	0.2410	0.8706
TS		0.00155	1480	0.000340	0.0666	0.0656	0.3106	0.9750	0.000249	0.5038	0.1675	0.2506	1446	0.0730	0.0635	0.2500
OCD		5.00E-05	73	0.000889	-0.1072	0.0656	0.1020	0.6945	0.000083	0.7009	0.1048	0.2729	71	-0.0956	0.0638	0.1336
AN		0.00215	1609	0.001183	0.1223	0.0648	0.0592	0.5801	0.000750	0.2543	-0.2893	0.2538	1757	-0.0104	0.1924	0.9569
ADHD	HD&MD	0.1331	45871	0.002017	0.1414	0.0370	1.34E-04	**2.70E-03**	0.001030	**0.0191**	0.1889	0.0806	44519	0.1493	0.0336	**8.94E-06**
ASD		0.04765	24772	0.000872	-0.0927	0.0369	0.0119	0.1478	0.000022	0.7338	0.0276	0.0813	26252	-0.0543	0.0561	0.3328
MDD		0.2014	74565	0.000854	0.0908	0.0365	0.0129	0.1708	0.000242	0.2544	0.0931	0.0817	77322	0.0912	0.0333	0.0062
SZ		0.0002	1906	0.000107	0.0331	0.0376	0.3781	0.9399	0.000069	0.5432	0.0496	0.0816	1695	0.0360	0.0341	0.2919
BIP		0.0063	7489	0.000323	-0.0567	0.0370	0.1256	0.6829	0.000215	0.2819	0.0860	0.0799	6569	-0.0029	0.0691	0.9661
TS		0.0037	3055	0.001259	0.1122	0.0372	0.0025	**3.93E-02**	0.000028	0.6978	-0.0321	0.0826	2677	0.0588	0.0695	0.3974
OCD		0.0005	428	0.000254	0.0495	0.0365	0.1749	0.8650	0.000020	0.7432	-0.0263	0.0803	374	0.0365	0.0332	0.2717
AN		0.0546	22200	0.000804	0.0899	0.0372	0.0157	0.2499	0.000103	0.4570	-0.0600	0.0807	21165	0.0320	0.0730	0.6606

Table S18. Two-sample Mendelian randomization analyses results for the significant polygenic correlations from PRS analysis. AD: Alcohol dependence, HD: heroin dependence, MD: MA dependence, HD&MD: combined HD and MD.

**Exposure**	**Outcome**	**Instrument variable**	**Methods**	**#SNP**	**B**	**SE (B)**	***P*-value**	**IVW regression test for heterogeneity**			**MR Egger test for pleiotropy**		
								**Q**	***Q df***	***P*-value**	**Egger intercept**	**SE**	***P*-value**
Risky drink	AD	SNPs with P<5e-8	**Inverse variance weighted**	**19**	**3.6106**	**0.5046**	**8.39E-13**	30.2521	18	**3.51E-02**	-0.0191	0.0407	0.6449
			MR Egger	19	**3.7628**	0.6094	1.02E-05						
			Weighted median	19	**3.6143**	0.4219	1.06E-17						
Risky sexual	MD	SNPs with P<5e-8	**Inverse variance weighted**	**48**	**1.8529**	**0.6959**	**7.75E-03**	74.2658	47	**6.82E-03**	-0.0692	0.0724	0.3442
			MR Egger	48	5.8652	4.2554	0.1748						
			Weighted median	48	**2.9723**	0.8768	6.99E-04						
Risky sexual	HD&MD	SNPs with P<5e-8	**Inverse variance weighted**	**44**	**1.5731**	**0.671**	**0.0191**	72.834	43	**3.01E-03**	-0.0467	0.0701	0.5091
			MR Egger	44	4.2733	4.1103	0.3045						
			Weighted median	44	1.9277	0.8003	0.016						
Risky smoke	MD	SNPs with P<5e-8	**Inverse variance weighted**	**70**	**1.7471**	**0.6372**	**6.11E-03**	113.3144	69	**6.19E-04**	-0.0037	0.0429	0.9315
			MR Egger	70	1.9845	2.826	0.4849						
			Weighted median	70	2.4671	0.7909	1.81E-03						
Risky smoke	HD&MD	SNPs with P<5e-8	**Inverse variance weighted**	**72**	**1.7858**	**0.5668**	**1.63E-03**	115.7358	71	**6.35E-04**	-0.0047	0.0342	0.89
			MR Egger	72	2.0856	2.2333	0.3536						
			Weighted median	72	2.0844	0.7345	4.55E-03						
EA	HD	SNPs with P<5e-8	**Inverse variance weighted**	**164**	**-2.7279**	**0.6677**	**4.39E-05**	285.5469	163	**9.71E-09**	-0.0417	0.0372	0.265
			MR Egger	164	0.1981	2.6995	0.9416						
			Weighted median	164	-4.7433	0.8386	1.55E-08						
EA	MD	SNPs with P<5e-8	**Inverse variance weighted**	**167**	**-2.2593**	**0.4849**	**3.17E-06**	261.431	166	**3.22E-06**	-0.033	0.0278	0.2368

			MR Egger	167	0.1187	2.0604	0.9541						
			Weighted median	167	-2.8888	0.6232	3.57E-06						
EA	HD&MD	SNPs with P<5e-8	**Inverse variance weighted**	**154**	**-2.2946**	**0.4747**	**1.34E-06**						
			MR Egger	154	-0.4216	2.1104	0.8419	249.8196	153	**1.26E-06**	-0.0259	0.0284	0.3638
			Weighted median	154	-3.3359	0.6024	3.07E-08						
CP	HD	SNPs with P<5e-8	**Inverse variance weighted**	**68**	**-1.6493**	**0.5836**	**4.71E-03**						
			MR Egger	68	1.5865	2.6674	0.554	96.3792	67	**1.08E-02**	-0.0694	0.0558	0.2183
			Weighted median	68	**-2.1031**	0.7467	4.85E-03						
CP	MD	SNPs with P<5e-8	**Inverse variance weighted**	**63**	**-1.7538**	**0.4879**	**3.25E-04**						
			MR Egger	63	-0.1057	2.6849	0.9687	94.4661	62	**4.95E-03**	-0.0339	0.0543	0.5347
			Weighted median	63	-2.3861	0.6029	7.57E-05						
CP	HD&MD	SNPs with P<5e-8	**Inverse variance weighted**	**65**	**-1.6827**	**0.4461**	**1.62E-04**						
			MR Egger	65	0.0155	2.368	0.9948	100.8282	64	**2.27E-03**	-0.0353	0.0483	0.4679
			Weighted median	65	**-1.6764**	0.5679	3.16E-03						
IQ	HD	SNPs with P<5e-8	Inverse variance weighted	81	-0.9301	0.6523	0.1539						
			MR Egger	81	1.5008	3.3056	0.6511	159.7978	80	**2.96E-07**	-0.0496	0.0661	0.4554
			Weighted median	81	**-2.058**	0.7885	9.06E-03						
IQ	MD	SNPs with P<5e-8	**Inverse variance weighted**	**85**	**-0.9548**	**0.4842**	**0.0486**						
			MR Egger	85	1.5038	2.7589	0.5872	156.2907	84	**2.87E-06**	-0.0487	0.0537	0.368
			Weighted median	85	-1.2106	0.5755	0.0354						
IQ	HD&MD	SNPs with P<5e-8	**Inverse variance weighted**	**76**	**-1.429**	**0.4824**	**3.06E-03**						
			MR Egger	76	2.0958	2.9711	0.4828	147.3015	75	**1.26E-06**	-0.0691	0.0575	0.2331
			Weighted median	76	**-1.5196**	0.584	9.27E-03						
ADHD	HD	SNPs with P<5e-8	**Inverse variance weighted**	**4**	**1.0355**	**0.4215**	**0.014**	0.5074	3	0.9173	-0.0163	0.217	0.947

			MR Egger	4	1.2013	2.2487	0.6466						
			Weighted median	4	1.0652	0.4737	0.0245						
ADHD	MD	SNPs with P<5e-8	Inverse variance weighted	3	0.3655	0.4609	0.4278						
			MR Egger	3	-0.2602	13.1921	0.9874	1.4018	2	0.4961	0.0478	1.0078	0.9698
			Weighted median	3	0.5139	0.5544	0.354						
ADHD	HD&MD	SNPs with P<5e-8	**Inverse variance weighted**	3	0.6758	0.3537	0.0561						
			MR Egger	3	1.714	1.8552	0.5252	0.337	2	0.8449	-0.0924	0.1621	0.6701
			Weighted median	3	0.6596	0.4298	0.1248						
ADHD	HD	SNPs with P<e-6	**Inverse variance weighted**	**17**	**0.6404**	**0.2986**	**0.032**						
			MR Egger	17	0.8077	1.5765	0.6159	24.2511	16	0.0842	0.0137	0.1265	0.9153
			Weighted median	17	0.9898	0.3496	0.0046						
ADHD	MD	SNPs with P<e-6	**Inverse variance weighted**	15	0.3885	0.2101	0.0644						
			MR Egger	15	-2.1328	1.5428	0.1901	7.9109	14	0.8939	-0.1901	0.1152	0.123
			Weighted median	15	0.5447	0.2662	0.0407						
ADHD	HD&MD	SNPs with P<e-6	**Inverse variance weighted**	**15**	**0.4223**	**0.1921**	**0.0279**						
			MR Egger	15	0.402	1.1175	0.7248	15.4346	14	0.3491	-0.0016	0.0868	0.9856
			Weighted median	15	0.6155	0.2558	0.0161						

References

1. Sun Y, Chang S, Liu Z, Zhang L, Wang F, Yue W, et al. (2021): Identification of novel risk loci with shared effects on alcoholism, heroin, and methamphetamine dependence. *Mol Psychiatry*. 26:1152-1161.
2. Purcell S, Neale B, Todd-Brown K, Thomas L, Ferreira MA, Bender D, et al. (2007): PLINK: a tool set for whole-genome association and population-based linkage analyses. *Am J Hum Genet*. 81:559-575.
3. Howie B, Marchini J, Stephens M (2011): Genotype imputation with thousands of genomes. *G3*. 1:457-470.
4. Delaneau O, Marchini J, Zagury JF (2012): A linear complexity phasing method for thousands of genomes. *Nat Methods*. 9:179-181.
5. Genomes Project C, Abecasis GR, Auton A, Brooks LD, DePristo MA, Durbin RM, et al. (2012): An integrated map of genetic variation from 1,092 human genomes. *Nature*. 491:56-65.
6. Chen J, Zheng H, Bei JX, Sun L, Jia WH, Li T, et al. (2009): Genetic structure of the Han Chinese population revealed by genome-wide SNP variation. *Am J Hum Genet*. 85:775-785.
7. Patterson N, Price AL, Reich D (2006): Population structure and eigenanalysis. *PLoS Genet*. 2:e190.
8. Price AL, Patterson NJ, Plenge RM, Weinblatt ME, Shadick NA, Reich D (2006): Principal components analysis corrects for stratification in genome-wide association studies. *Nat Genet*. 38:904-909.
9. Willer CJ, Li Y, Abecasis GR (2010): METAL: fast and efficient meta-analysis of genomewide association scans. *Bioinformatics*. 26:2190-2191.
10. Bulik-Sullivan B, Finucane HK, Anttila V, Gusev A, Day FR, Loh PR, et al. (2015): An atlas of genetic correlations across human diseases and traits. *Nat Genet*. 47:1236-1241.
11. Watanabe K, Taskesen E, van Bochoven A, Posthuma D (2017): Functional mapping and annotation of genetic associations with FUMA.

Nature communications. 8:1826.
12. Consortium GT (2015): Human genomics. The Genotype-Tissue Expression (GTEx) pilot analysis: multitissue gene regulation in humans. *Science*. 348:648-660.
13. Glasser MF, Sotiropoulos SN, Wilson JA, Coalson TS, Fischl B, Andersson JL, et al. (2013): The minimal preprocessing pipelines for the Human Connectome Project. *Neuroimage*. 80:105-124.
14. Smith SM, Beckmann CF, Andersson J, Auerbach EJ, Bijsterbosch J, Douaud G, et al. (2013): Resting-state fMRI in the Human Connectome Project. *Neuroimage*. 80:144-168.
15. de Leeuw CA, Mooij JM, Heskes T, Posthuma D (2015): MAGMA: generalized gene-set analysis of GWAS data. *PLoS computational biology*. 11:e1004219.
16. Pinero J, Bravo A, Queralt-Rosinach N, Gutierrez-Sacristan A, Deu-Pons J, Centeno E, et al. (2017): DisGeNET: a comprehensive platform integrating information on human disease-associated genes and variants. *Nucleic Acids Res*. 45:D833-D839.
17. Zhou Y, Zhou B, Pache L, Chang M, Khodabakhshi AH, Tanaseichuk O, et al. (2019): Metascape provides a biologist-oriented resource for the analysis of systems-level datasets. *Nature communications*. 10:1523.
18. Tobacco, Genetics C (2010): Genome-wide meta-analyses identify multiple loci associated with smoking behavior. *Nat Genet*. 42:441-447.
19. Zhou H, Sealock JM, Sanchez-Roige S, Clarke TK, Levey DF, Cheng Z, et al. (2020): Genome-wide meta-analysis of problematic alcohol use in 435,563 individuals yields insights into biology and relationships with other traits. *Nature neuroscience*. 23:809-818.
20. Kranzler HR, Zhou H, Kember RL, Vickers Smith R, Justice AC, Damrauer S, et al. (2019): Genome-wide association study of alcohol consumption and use disorder in 274,424 individuals from multiple populations. *Nature communications*. 10:1499.

21. Zhou H, Rentsch CT, Cheng Z, Kember RL, Nunez YZ, Sherva RM, et al. (2020): Association of OPRM1 Functional Coding Variant With Opioid Use Disorder: A Genome-Wide Association Study. *JAMA psychiatry*. 77:1072-1080.
22. Karlsson Linner R, Biroli P, Kong E, Meddens SFW, Wedow R, Fontana MA, et al. (2019): Genome-wide association analyses of risk tolerance and risky behaviors in over 1 million individuals identify hundreds of loci and shared genetic influences. *Nat Genet*. 51:245-257.
23. Lee JJ, Wedow R, Okbay A, Kong E, Maghzian O, Zacher M, et al. (2018): Gene discovery and polygenic prediction from a genome-wide association study of educational attainment in 1.1 million individuals. *Nat Genet*. 50:1112-1121.
24. Savage JE, Jansen PR, Stringer S, Watanabe K, Bryois J, de Leeuw CA, et al. (2018): Genome-wide association meta-analysis in 269,867 individuals identifies new genetic and functional links to intelligence. *Nat Genet*. 50:912-919.
25. Levey DF, Stein MB, Wendt FR, Pathak GA, Zhou H, Aslan M, et al. (2021): Bi-ancestral depression GWAS in the Million Veteran Program and meta-analysis in >1.2 million individuals highlight new therapeutic directions. *Nature neuroscience*. 24:954-963.
26. Euesden J, Lewis CM, O'Reilly PF (2015): PRSice: Polygenic Risk Score software. *Bioinformatics*. 31:1466-1468.
27. Burgess S, Scott RA, Timpson NJ, Davey Smith G, Thompson SG, Consortium E-I (2015): Using published data in Mendelian randomization: a blueprint for efficient identification of causal risk factors. *European journal of epidemiology*. 30:543-552.
28. Hemani G, Zheng J, Elsworth B, Wade KH, Haberland V, Baird D, et al. (2018): The MR-Base platform supports systematic causal inference across the human phenome. *Elife*. 7.
29. International Consortium for Blood Pressure Genome-Wide Association S, Ehret GB, Munroe PB, Rice KM, Bochud M, Johnson AD, et al. (2011): Genetic variants in novel pathways influence blood pressure and cardiovascular disease risk. *Nature*. 478:103-109.
30. Martin AR, Kanai M, Kamatani Y, Okada Y, Neale BM, Daly MJ (2019): Clinical use of current polygenic risk scores may exacerbate health disparities. *Nat Genet*. 51:584-591.
